# SeDeM tool-driven full factorial design for osmotic drug delivery of tramadol HCl: Formulation development, physicochemical evaluation, and *in-silico* PBPK modeling for predictive pharmacokinetic evaluation using GastroPlus™

**DOI:** 10.3389/fphar.2022.974715

**Published:** 2022-10-07

**Authors:** Muhammad Talha Saleem, Muhammad Harris Shoaib, Rabia Ismail Yousuf, Farrukh Rafiq Ahmed, Kamran Ahmed, Fahad Siddiqui, Zafar Alam Mahmood, Muhammad Sikandar, Muhammad Suleman Imtiaz

**Affiliations:** Department of Pharmaceutics, Faculty of Pharmacy and Pharmaceutical Sciences, University of Karachi, Karachi, Sindh, Pakistan

**Keywords:** tramadol, SeDeM diagram expert system, PBPK, osmotic tablet, controlled release, gastroplus

## Abstract

The study is based on using SeDeM expert system in developing controlled-release tramadol HCl osmotic tablets and its *in-silico* physiologically based pharmacokinetic (PBPK) modeling for *in-vivo* pharmacokinetic evaluation. A Quality by Design (QbD) based approach in developing SeDEM-driven full factorial osmotic drug delivery was applied. A 2^4^ Full-factorial design was used to make the trial formulations of tramadol HCl osmotic tablets using NaCl as osmogen, Methocel K4M as rate controlling polymer, and avicel pH 101 as diluent. The preformulation characteristics of formulations (F1-F16) were determined by applying SeDeM Expert Tool. The formulation was optimized followed by *in-vivo* predictive pharmacokinetic assessment using PBPK “ACAT” model of GastroPlus™. The FTIR results showed no interaction among the ingredients. The index of good compressibility (ICG) values of all trial formulation blends were ≥5, suggesting direct compression is the best-suited method. Formulation F3 and F4 were optimized based on drug release at 2, 10, and 16 h with a zero-order kinetic release (*r* ^2^ = 0.992 and 0.994). The SEM images confirmed micropores formation on the surface of the osmotic tablet after complete drug release. F3 and F4 were also stable (shelf life 29.41 and 23.46 months). The *in vivo* simulation of the pharmacokinetics of the PBPK *in-silico* model revealed excellent relative bioavailability of F3 and F4 with reference to tramadol HCl 50 mg IR formulations. The SeDeM expert tool was best utilized to evaluate the compression characteristics of selected formulation excipients and their blends for direct compression method in designing once-daily osmotically controlled-release tramadol HCl tablets. The *in-silico* GastroPlus™ PBPK modeling provided a thorough pharmacokinetic assessment of the optimized formulation as an alternative to tramadol HCl *in vivo* studies.

## Introduction

An elementary osmotic pump (EOP) is the simplest form of osmotic drug delivery system that consists of the combination of active pharmaceutical ingredients and excipients present within the core with or without an osmotic agent. The core system is externally covered with a non-extensible semipermeable membrane and a drilled orifice for drug release (Stuti et al., 2011). The gastrointestinal fluid imbibes within the core due to the osmotic pressure difference across the semipermeable membrane. Consequently, the release of the drug through the orifice takes place. Various formulation factors affect drug release from EOP like membrane thickness, orifice diameter, amount of plasticizer, and the concentration of osmotic agents. The EOP is a suitable dosage form for designing controlled release formulation of drugs like tramadol HCl having moderate to high solubility (Verma et al., 2000).

The SeDeM expert tool is diagrammatically used to assess the compressibility of pharmaceutical powder excipients and its suitability for commercial-scale tablet manufacturing by the direct compression method (Pérez et al., 2006). This method uses mathematical transformations on 12 flow and compressibility-related characteristics of the powders to generate a unique numerical and graphical profile on a scale of 0–10. Moreover, it also indicates the inherent deficiencies of active pharmaceutical ingredients (APIs) that could be overcome by adding appropriate excipients in required ratios. This system reduces the number of trials and time needed to develop an optimized formulation for commercial tablet manufacturing by direct compression (Suñé-Negre et al., 2008).

The “Advanced Compartmental and Transit” (ACAT) model in PBPK modeling is an approach for determining the pharmacokinetics of drugs in a mechanistic manner. Various biopharmaceutics parameters such as physicochemical properties of the drug molecules (pKa, Log P, solubility, particles size, etc.), physiological conditions related to absorption (gastrointestinal pH, gut and tissues spaces, perfusion rate, etc.), and drug pharmacokinetics (volume of distribution, clearance, rate constants, etc.) are processed to predict *in-vivo* pharmacokinetics and performance of formulated drugs (Kuentz et al., 2006).

Tramadol HCl is one of the generally safe opioid analgesics with a low potential for dependence. It is used in the pain management of osteoarthritis alone or combination with other analgesics (Scott and Perry, 2000). Compared to other opioid analgesics, tramadol HCl does not cause respiratory distress and gastrointestinal irritation. There are many drug delivery systems designed and available for tramadol used for different types of delivery. Usually, it is given 50 mg after every 4–6 h to manage chronic pain (Wiffen et al., 2017). The absorption of tramadol is complete and relative bioavailability is about 70% because of first-pass metabolism (Eassa and El-Shazly, 2013; Vazzana et al., 2015). Due to its high dosing frequency, tramadol HCl is a good candidate for controlled release formulation. It is a BCS class-I drug; therefore, designing sustained release formulations requires a higher proportion of matrix-based polymers (Klančar et al., 2015). The elementary osmotic pumps for such highly soluble drugs reduce the polymer concentration and help release the drug at zero order. This can be achieved by the combined effect of osmogen, orifice size on the surface of tablets, and different polymers (Prabakaran et al., 2003).

Though tramadol HCl is available as a controlled release system prepared by different techniques, no single comprehensive study was designed on an osmotic system with precision and accuracy with a robust zero-order release for this drug. The present work is based on using the SeDeM expert system for the first time in designing once-daily controlled-release osmotic tablets by direct compression method taking tramadol HCl as a model drug. The *in-silico* ACAT (PBPK) simulation, using GastroPlusTM, was also evaluated for estimating plasma drug concentration-time profiles of the optimized formulations.

## Experimental

Tramadol HCl (% purity = >99.8%) was gifted from Atco Pharmaceuticals (Karachi, Pakistan). Opadry^®^ CA (fully formulated osmotic coating system composed of cellulose acetate as water insoluble component and polyethylene glycol (PEG) as pore-former) and Methocel™ K4M Premium grade were provided by Colorcon Limited (Kent, United Kingdom). Avicel™ PH-101, Magnesium Stearate, and Aerosil™ 200 were purchased from FMC Corporation (United States). Sodium Chloride [≥99.5% w/w], Potassium dihydrogen Phosphate [≥99.5% w/w], sodium hydroxide [≥99% w/w] and hydrochloric acid [37–38% w/w] were purchased from Merck (Germany).

### Design of experiments (DoE) for elementary osmotic tablets

A four-factor two-level factorial design using the Design Expert Software (Version 10, Stat-Ease, Minneapolis, United States) was applied in a randomized order to understand the main effect and interaction effect of all factors at each level (see Table 1). The independent variables were NaCl (X1) as an osmotic agent, Methocel™ K4M (X2) as a release rate controlling agent, percent weight gain after coating with Opadry^®^ CA (X3), and orifice diameter (X4), which were set at low and high levels coded as +1 and −1 respectively. The selection of in put variables and their level ranges were based on preliminary reported studies (El-Zahaby et al., 2018; Farooqi et al., 2020), so that their influence on critical response variables, drug release (%) at 2 h (Y1), 12 h (Y2), 16 h (Y3), and RSQ_zero_ (Y4) can be studied (Shah and Prajapati, 2019). A stepwise regression analysis was conducted using a full factorial design to determine the influence of significantly controlling input factors on response variables (Xue et al., 2015). A two-way analysis of variance (ANOVA) was performed to ascertain the reliability of the findings. The model was considered significant at a 5% level of significance (*p* ≤ 0.05). The influence of independent variables on critical responses was graphically illustrated by perturbation plots and 3D response surface curves. For each critical response model equation was generated for the prediction of the optimized formulation. The percentages of Aerosil^®^ 200 and magnesium stearate were kept fixed in each formulation as 0.5 and 1.5%, respectively. Trial formulations (F1-F16) and pre-compression characteristics were evaluated using the SeDeM expert tool.

**TABLE 1:** Selection of factors, levels, and responses for 2^4^ full factorial design.

Independent variables	Levels	
	Low	High
X_1_ = Concentration of Osmogen (%)	4	8
X_2_ = Concentration of Methocel™ ^®^ K4M (%)	10	20
X_3_ = Coating Weight Gain (%)	8	12
X_4_ = Orifice Diameter (mm)	0.2	0.8
**Dependent Variables**		**Constraints**
	Y_1_ = Cumulative % drug release in 2 h	0% < Y_1_ < 15%
	Y_2_ = Cumulative % drug release in 12 h	65% < Y_2_ <95%
	Y_3_ = Cumulative % drug release in 16 h	80% < Y_3_ < 110%
	Y_4_ = *r* ^2^ (RSQ Zero)	Y_4_ Maximum (>0.9)

### Preformulation evaluation by SeDeM expert tool

All powders ingredients, including drug, excipients and, formulation blends (F1-F16) were evaluated for their micromeritic-based properties, and the following 12 SeDeM parameters were estimated by following pharmacopial methods, to ascertain their use as suitable exepients for direct compression (see Table 2 for SeDeM parameters) (U.S.P.38-N.F.33, 2014; Ph.Eur.10, 2019).

**TABLE 2:** SeDeM Parameters, indices, limit values and calculating factors.

Incidence	Parameters	Limit value (*v*)	Factors applied to *v*	Radius (*r)*
Dimension	Bulk density (Da)	0–1 g/ml	10*v*	0–10
	Tapped density (Dc)	0–1 g/ml	10*v*	0–10
Compressibility	Inter-particle porosity (Ie)	0–1.2	10*v*/1.2	0–10
	Carr’s index (IC)	0–50 (%)	*v*/5	0–10
	Cohesion index (Icd)	0–200 (N)	*v*/20	0–10
Flowability/powder flow	Hausner’s ratio (IH)	1–3	5 (3 - v)	0–10
	Angle of repose (α)	50–0 (°)	10 - (*v*/5)	0–10
	Powder flow (t^n^)	20–0 (s)	10 - (*v*/2)	0–10
Lubricity/Stability	Loss on drying (% HR)	0–10 (%)	10 - *v*	0–10
	Hygroscopicity (% H)	20–0 (%)	10 - (*v*/2)	0–10
Lubricity/dosage	Particle size (% Pf)	50–0 (%)	10 - (*v*/5)	0–10
	Homogeneity index (Iθ)	0–2 × 10^–2^	500*v*	0–10

#### Bulk Density (Da).

The density measurement instrument VTAP/MATIC-II (Veego Instruments, Mumbai, India) was used to measure bulk density, tapped density, Hausner’s ratio, and compressibility index, using 10 gm of each material. Various parameters were calculated using the following formulae:

$$Da=P/V_{a}$$ (1)

Where “*V*_*a*_”is the volume occupied by the powder in a cylinder for a given weight of the powder “*P*”.

#### Tapped Density (Dc)

$$Dc=P/V_{c}$$ (2)

Where “*V*_*c*_” is the volume occupied by the powder in the cylinder after 2500 taps for a given weight of powder “*P*”.

#### Inter-particle Porosity (Ie)

Ie=Dc−Da/Dc x Da (3)

#### Carr’s Index (IC)

IC=(Dc−Da/Dc)×100 (4)

#### Hausner’s Ratio (IH)

IH=Dc/Da (5)

#### Cohesion Index (Icd)

This parameter indicates the presence of strong, cohesive forces between powder particles when a powder material is subjected to high compression force. In this test, the powder is subjected to high compression force to produce compressed tablets of a pre-determined size, using an eccentric single punch tablet press (Korsch, Frankfurt, Germany). The hardness of prepared compressed tablets was evaluated using Dr. Schleuniger Multitest 50 (Pharmatron, Switzerland), and an average value of 3 tablets was calculated in Newton (N) (Neǵre et al., 2013).

#### Angle of Repose (α).

To determine the angle of repose, powder material was allowed to pass through the funnel of 9.5 cm height, with upper spout and internal orifice diameters of 7.2 and 1.8 cm, respectively. The funnel was mounted at about 20 cm height from the base of the Petri dish and placed on the working bench. The material was filled in the funnel with plugged spout, then allowed to flow through the orifice and collected in a Petri dish. The diameter “*d*” of the cone and its height “*h*” was measured thrice with the help of a sliding Vernier caliper. The mean values of ‘d’ and ‘h’ were used to calculate the angle of repose by the given formula.

tan⁡∝=2h/d (6)

#### Flowability (t”)

Following the method given in the European Pharmacopeia, flowability of the powder blends were determined through the same funnel used to measure the angle of repose (Ph.Eur.10, 2019). A powder sample of 100 g was loaded in the funnel, and the time taken by the whole powder to pass through the orifice, was recorded using an electronic stopwatch. The test was performed in triplicate, and the mean value was calculated.

#### Loss on drying (%HR)

Loss on drying was determined by taking 2 g of the powders individually and their blends in a Petri dish, and the samples were dried in a hot air oven by forcing circulation of air at 105°C for 2 h. The weight of the samples were taken at regular intervals until a constant weight was obtained, and finally, the percent weight loss from the sample was calculated.

#### Hygroscopicity (%H)

The powder samples were taken in separate Petri dishes and placed in a humidifier maintained at a temperature of 22 ± 2°C with a relative humidity of about 76 ± 2%. The percentage mass gained by the samples after 24 h was calculated.

#### Particle size under 50 μm (%Pf)

A random sample of 100 g of powder was placed on a gyratory shaker mounted with a standard sieve of 0.05 mm. The powders were sieved for 10 min, and the cumulative weight of undersize particles was measured. The average value was calculated after conducting the test three times.

#### Homogeneity index (Iθ)

To determine the uniformity in the particle size, a gyratory shaker was mounted with sieves of different sizes in a series of 355, 212, 100, and 50 μm. A sample weight of 100 g was placed on the uppermost sieve and was subjected to sieving for 10 min. The mean value from the triplicate of the test was used to calculate the homogeneity index using the following formula.

$$I\theta =\frac{Fm}{\begin{array}{c} 100+\left(dm-dm_{-1}\right)\;Fm_{-1}+\left(dm_{+1}-dm\right)\;Fm_{+1}+\\ \left(dm-dm_{-2}\right)Fm_{-2}+\left(dm_{+2}-dm\right)Fm_{+2}+\ldots ..\\ ....\left(dm-dm_{-n}\right)Fm_{-n}+\left(dm_{+n}-dm\right)\;Fm_{+n} \end{array}}$$ (7)

Where,

“Fm” and “dm” represents the percentage of particles in major size range and their average diameter, respectively.

“Fm-1” and “dm-1” represents the percentage of particles below the major size range and their average diameter, respectively.

“Fm+1” and “dm+1” represents the percentage of particles above the major size range and their average diameter, respectively.

“n” is the number of fractions taken in the series.

### SeDeM parametric indices

Using Table 2, the measured data values were converted into linear parametric values, and following SeDeM parametric indices were calculated.

$$a)\;Parameter\;Index\;\left(IP\right)=\frac{n{}^{\circ}\;P\geq 5}{n{}^{\circ}}\;(\geq 0.\;5)$$ (8)

Where n° P≥5 = the number of parameters with a value equal to or higher than 5 and n° = Total number of parameters studied

$$b)\;Paramter\;Profile\;Index\;\left(IPP\right)=Av.^{\prime }r^{\prime }of\;all\;n{}^{\circ}\;values\;(\geq 5)$$ (9)

Where the acceptance limit is: IPP = mean r ≥ 5

$$c)\;Reliability\;Factor\;\left(f\right)=\frac{Polygon\;Area}{Circle\;Area}$$ (10)

When the tested parameters are 12, *f* = 0.952

d) Good Compressibility Index (ICG)=IPP×f (≥5) (11)

After calculating all the 12 parametric indices, the data were analyzed by constructing a radar chart, and the final ICG value for each powder and blends (F1-F16) was calculated.

The lowest parametric incidence value was rectified by Avicel PH-101 and the minimum amount of Avicel PH-101 required for the correction was calculated using the given formula,

$$CP=100-\left[\left(\frac{RE-R}{RE-RP}\right)\times 100\right]$$ (12)

Where,

“*CP*” is the amount of corrective excipient required in percentage to overcome the deficient index of powder.

“*RE*” is the parametric incidence value of the corrective excipient used to overcome the deficient index of powder.

“*RP*” is the parametric incidence value of the powder needed to be corrected.

“*R*” is the minimum or desired value of the incidence parameter (at least 5) (Suñé-Negre et al., 2008; Aguilar-Díaz et al., 2012; Suñé-Negre et al., 2014).

### Preparation of core tablets

All the formulations were compressed into tablets by using the direct compression method. Crystalline ingredients of the formulations were crushed, and all the powders were separately sieved through a Mesh # 30 sieve. The calculated amounts of tramadol HCl and formulation excipients (Avicel™ PH-101, Methocel™ K4M and Sodium Chloride) were mixed by the tumbling action for 8–10 min (optimized blending time obtained during preliminary studies). Magnesium stearate and Aerosil™ 200 were also added and blended for 3 min further. The powder blend was compressed into tablets with the target compression weight of 500 mg using biconcave punches fixed in an eccentric single punch tablet press (Korsch, Frankfurt, Germany).

### Coating of tablet and orifice formation

After 48 h resting period, to ensure the completion of elastic stress relaxation time, the tablets were coated with a commercially available coating system Opadry^®^ CA to form a semipermeable membrane. The coating solution containing 7% w/w of Opadry^®^ CA, was prepared in a solvent system containing acetone and water (9:1) by weight (Ahmed et al., 2018). The compressed tablets were coated in a conventional tablet coating pan, which was rotated at a 3–6 rpm speed till the inlet and outlet air temperatures were maintained at ∼45 and ∼28°C, respectively (Okimoto et al., 2004; Seo et al., 2020). The speed of the coating pan was then increased up to 18–24 rpm (Lin et al., 2022). The automation pressure was set at 1 kg/cm^2^, and the coating solution was applied at a spraying rate of 8–10 ml/min. When the desired coating weight gain was attained, the coated tablets were dried at 50°C for16 h in a hot air oven. Micro-drilling bits of 0.2 and 0.8 mm were used to create an orifice in the center of one side of the tablet.

### Pharmaceutical quality evaluation of tablets

#### Physical evaluation

All the coated and core tablets were subjected to USP quality assessment tests. A random sample of 20 tablets from each formulation was taken individually, and weight variation was determined using a digital balance (Sartorius, Germany). The thickness and diameter of the tablets (*n* = 20) were measured by a digital vernier caliper (Seiko brand, China), and to determine the mechanical strength of core tablets, a hardness test was performed using Dr. Schleuniger Pharmatron M50 MultiTest 50 (Pharmatron, Switzerland) and percentage friability was calculated following USP method by using Roche type friabilitor (Erweka D2800, Heusenstamm, Germany) (U.S.P.38-N.F.33, 2014),

##### Content assay

The chemical assay of formulations was performed by the HPLC technique according to the method described in the USP. The standard solution was prepared in a 10 ml flask by dissolving 10 mg of tramadol HCl in a 20% flask volume of methanol and making up the volume with distilled water. The reference standard solution of 25 µg/ml tramadol HCl was prepared in the mobile phase containing tetrahydrofuran, trifluoroacetic acid, triethylamine, and water (10:0.1:0.1:90), and flown at a rate of 1 ml/min. The test solutions of the same strength were prepared by taking ten tablets of each trial batch. Chromatographic separation was performed on a C18 (3.9 × 300 mm, Bondapak RP column) at 25°C using HPLC (LC-10 AT VP Shimadzu Japan). The tramadol HCl response was recorded at 271 nm using a UV detector (LC 10a VP, Shimadzu, Japan).

##### *In vitro* drug release studies

The drug release studies of tramadol HCl from F1-F16 were conducted using USP Type-II apparatus (Erweka DT600, GmbH, Huesenstamm, Germany). The tablets were initially tested in 900 ml dissolution medium pH 1.2 followed by phosphate buffer pH 6.8 at the agitation speed of 75 rpm, and the medium temperature was maintained at 37 ± 0.5°C. A sample of 10 ml was drawn (replaced with fresh medium) and filtered, then a working concentration equivalent to 0.13 mg/ml was made in the dissolution medium pH 1.2 for 2 h. The procedure was continued for 24 h after replacing the dissolution medium, i.e., phosphate buffer pH 6.8. The cumulative percentage of drug release was determined by UV-spectrophotometer (UV-1800, Shimadzu Corporation, Kyoto, Japan) at 291 nm (Ahmed et al., 2018).

To study the mechanism of drug release, different kinetic models, first-order, zero-order, Higuchi, Korsmeyer Peppas, Hixon Crowell, Baker Lonsdale, and Weibull, were applied to analyze the kinetic mechanism of *in-vitro* drug release using DD-Solver (Costa and Lobo, 2001).

#### Experimental

##### Formulation optimization

Numerical optimization procedure using “Design Expert” software version 10 (Stat-Ease, Minneapolis, United States) was adopted to observe the applicability of the model equation and the perturbing effect of input variables for the prediction of targeted responses. The optimization criteria included compliance to multiple dissolution time points for the release of tramadol HCl at 2 (Y1), 12 (Y2), and 16 (Y3) hours and RSQ (Y4, coefficient of correlation for zero-order release). Based on the desirability value, formulations F3 and F4 were taken as the optimized formulations against the targeted constraints.

##### Effect of agitation rate and pH on drug release

The optimized formulations (F3 and F4) were subjected to *in-vitro* dissolution testing for the determination of the effect of varying agitation rates, 50, 75, and 100 rpm and pH (1.2, 4.5, and 6.8) on tramadol HCl release from osmotically controlled tablets. The turbulence created by the different rotational speeds of the paddle affects the drug release from the osmotic pump. The percentage of drug release was determined by the same method as reported in section 2.6.3. The dissolution profiles of optimized formulations were also compared statistically by applying the *f*_2_ similarity factor test using the given formula (Muselík et al., 2021):

$$f2=50\log\left\{{\left[1+\frac{1}{n}\;\overset{n}{\underset{n-1}{\sum }}{\left(R_{t}-T_{t}\right)}^{2}\right]}^{-0.5}\times 100\right\}$$ (13)

Where *n* is a number of time points and *Rt* and *Tt* are the mean percentages of the released drug from the (*R*) and (*T*) products, respectively, at the *t* time point, 1 ≤ *t* ≤ *n*.

##### Fourier transform infrared spectroscopy (FTIR) analysis

The FTIR method was used to detect any interaction between drugs and excipients. The pure drug and powder excipients in the ratio of 1:1 were subjected to Fourier transform infrared (FTIR) spectroscopy (Nicolet-6700; Thermo Scientific, United States), and the spectrum was generated from 4,000–400 cm^−1^. Similarly, the core tablets of the optimized formulations were crushed separately in a mortar and pestle and were also analyzed to observe any post-compression interaction. Finally, the IR spectra of the pure drug were compared with that of the samples.

##### Scanning electron microscopy analysis

The surface morphology of the optimized formulations was analyzed by SEM study before and after drug release. The SEM samples were prepared by mounting them on aluminum studs and then coated with gold up to 250°A. After 24 h of dissolution testing, the coated tablets were dried at 50°C for 12 h in a hot air oven before microscopy (JSM-6380A, JEOL, Japan).

##### Stability studies

The optimized formulations were packed in plastic-lined tight amber glass bottles and were subjected to accelerated stability conditions for 0, 1, 3, and 6 months at 40 ± 2°C and RH of 75 ± 5% in the stability chamber (NuAire, Plymouth, MN, United States) as per ICH guidelines. Various pharmaceutical quality characteristics, content assay, and dissolution profiles of the formulations were determined at 0, 1, 3, and 6 months as per ICH guidelines (ICH Harmonized Tripartite, 2003) Q1A (R2). The shelf-life of the optimized formulation was computed by Minitab 17 software (Minitab, Pennsylvania, United States).

##### *In silico* simulation of tramadol HCl by PBPK model

The drug release data of the optimized formulations of tramadol HCl (F3 and F4; 190 mg) were subjected to *in-silico* “Physiologically Based Pharmacokinetics” (PBPK) modeling and simulation using “Advanced Compartmental and Transit” (ACAT) model embedded in GastroPlus™ software version 9.8 (Simulations Plus Inc., Lancaster, CA, United States). Log *p* values, molecular weight, diffusion coefficient, drug particle density, jejunal effective permeability, and human blood-plasma concentration ratio of tramadol HCl were calculated and acquired from the ADMET™ predictor (in GastroPlus™) module. The 2-compartmental pharmacokinetics values of tramadol HCl, such as *V*_*c*_, *K*_*12*_ *, K*_*21,*_ and *C*_*L*_ were calculated after processing and modeling (compartmental modeling) the reported *in-vivo* pharmacokinetics data of immediate-release (IR) tramadol HCl 50 mg tablets by Brvar et al., 2014, in PKPlus™ module of GastroPlus™ (Brvar et al., 2014). A multiple doses (50 mg q6h for 1 day) pharmacokinetic profile was also constructed using the same data, and values such as *C*_max_ *, T*_max_ *, AUC*_*t*_ *, AUC*_*inf.*_ were calculated. Moreover, the values of *pKa*, aqueous solubility, and unbound fraction of the drug in plasma were obtained from the reported literature (Tetko et al., 2001; T’jollyn et al., 2015; T’jollyn et al., 2018). The description of these input parameters for the ACAT model is given in Table 3. Using these values as input, simulated *in-vivo* drug concentration profiles were constructed for the *in-vitro* release data of optimized formulations (F3 and F4) in the 2-stage media release and drug release media with three different pH values (1.2, 4.5, and 6.8). Finally, the pharmacokinetic profiles of the optimized formulations were compared with that of multiple-dose IR tramadol tablets following the methodology reported in the literature, and relative bioavailability for optimized formulations was calculated (Wang et al., 2015; Pilla Reddy et al., 2018).

**TABLE 3:** Parameters and values for ‘Advanced Compartment and Transit (ACAT) modeling in GastroPlus™

Parameter	Value	Source
*Log P*	2.64	ADMET Predictor™
*pKa*	9.2	(11)
Molecular weight (*MW*) (g/mol)	263.38	ADMET Predictor™
Aqueous solubility (*S*) (mg/ml)	0.75	(12)
Diffusion coefficient (*D*) (cm^2^/sec × 10^–5^)	0.75	ADMET Predictor™
Drug particle density (g/ml)	1.2	ADMET Predictor™
Jejunal effective permeability (*P*_*eff*_) (cm/sec ×10^–4^)	3.26	ADMET Predictor™
Unbound percent in human plasma (% F_up_)	75–80	(13)
Human blood to plasma concentration ratio (R_bp_)	1.03	ADMET Predictor™
*V*_*c*_ (L/kg)	1.4776	PKPlus™
*K*_*12*_ (L/h)	0.4021	PKPlus™
*K*_*21*_ (L/h)	0.0569	PKPlus™
Clearance (*C*_*L*_) (L/h/kg)	0.00572	PKPlus™

## Results

### Design of experiments (DoE) for elementary osmotic tablets

A multivariate 2^4^ full factorial design was successfully applied to control the release of tramadol HCl for 24 h from osmotically controlled tablets. A total of 16 formulations containing 190 mg of tramadol HCl were prepared (Table 4).

**TABLE 4:** Composition of tramadol HCl formulations.

Formulations code	Tramadol HCl		Avicel pH 101^®^		Sodium chloride		Methocel™ K4M		Magnesium stearate		Aerosil™ 200		Tablet Weight	Coating gain	Orifice size
	Drug		Filler/Binder		Osmogen		Release retardant		Lubricant		Glidant				
	mg	%	mg	%	mg	%	mg	%	mg	%	mg	%	mg	%	mm
F1	190	38	230	46	20	4	50	10	7.5	1.5	2.5	0.5	500	8	0.2
F2	190	38	230	46	20	4	50	10	7.5	1.5	2.5	0.5	500	8	0.8
F3	190	38	230	46	20	4	50	10	7.5	1.5	2.5	0.5	500	12	0.2
F4	190	38	230	46	20	4	50	10	7.5	1.5	2.5	0.5	500	12	0.8
F5	190	38	210	42	40	8	50	10	7.5	1.5	2.5	0.5	500	8	0.2
F6	190	38	210	42	40	8	50	10	7.5	1.5	2.5	0.5	500	8	0.8
F7	190	38	210	42	40	8	50	10	7.5	1.5	2.5	0.5	500	12	0.2
F8	190	38	210	42	40	8	50	10	7.5	1.5	2.5	0.5	500	12	0.8
F9	190	38	180	36	20	4	100	20	7.5	1.5	2.5	0.5	500	8	0.2
F10	190	38	180	36	20	4	100	20	7.5	1.5	2.5	0.5	500	8	0.8
F11	190	38	180	36	20	4	100	20	7.5	1.5	2.5	0.5	500	12	0.2
F12	190	38	180	36	20	4	100	20	7.5	1.5	2.5	0.5	500	12	0.8
F13	190	38	160	32	40	8	100	20	7.5	1.5	2.5	0.5	500	8	0.2
F14	190	38	160	32	40	8	100	20	7.5	1.5	2.5	0.5	500	8	0.8
F15	190	38	160	32	40	8	100	20	7.5	1.5	2.5	0.5	500	12	0.2
F16	190	38	160	32	40	8	100	20	7.5	1.5	2.5	0.5	500	12	0.8

### Pre-formulation evaluation by SeDeM expert tool

**FIGURE 1:**
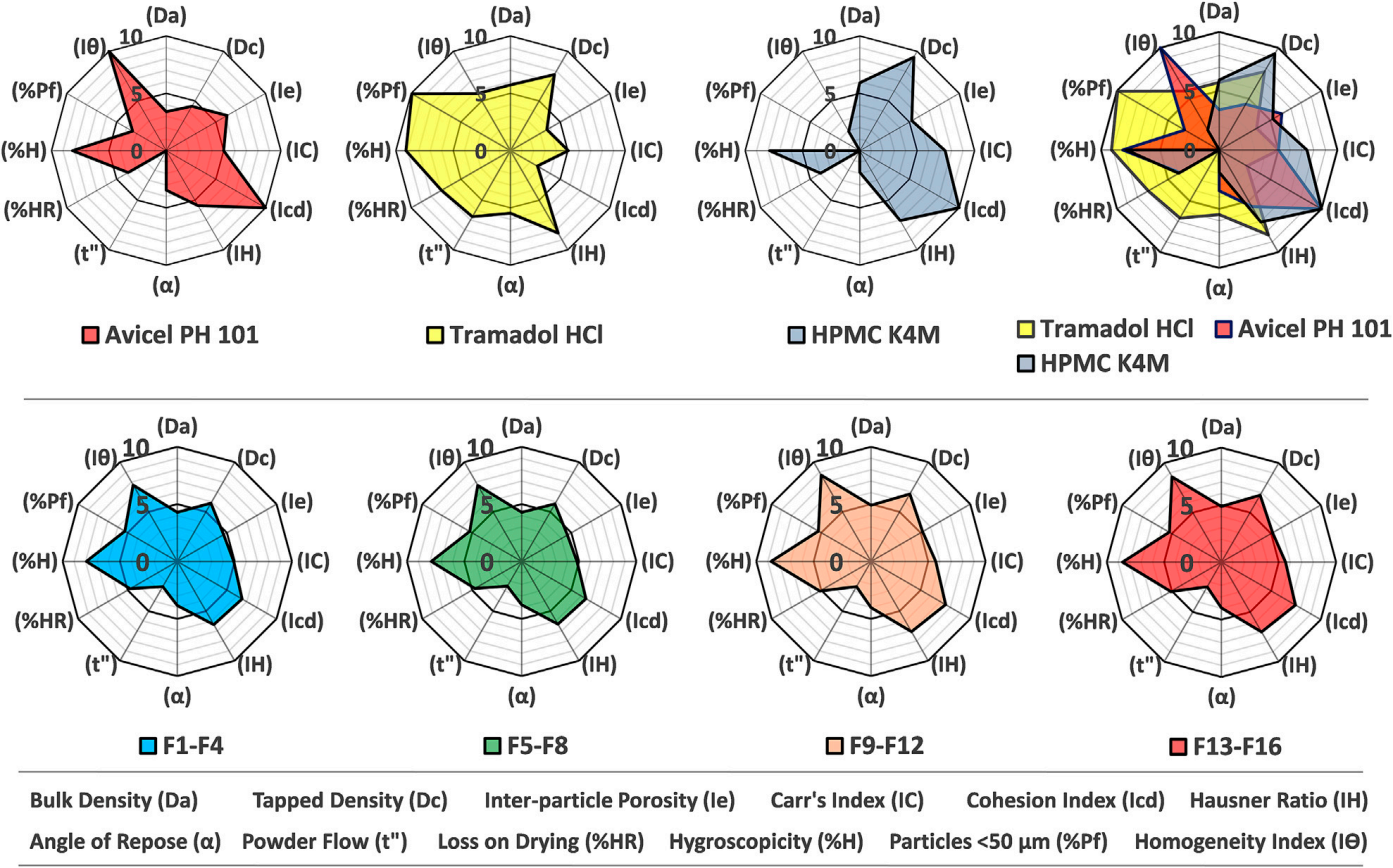
SeDeM diagram (radar plot) of Avicel PH101, tramadol HCl, Methocel™ K4M, overlay plot (upper segment) and formulations from F1-F4, F5-F8, F9-F12 and F13-F16 (lower segment).

The results of the SeDeM tool illustrated that all the ingredients exhibited satisfactory compressional characteristics, and the final Index of Good Compressibility (IGC) value of tramadol HCl was 6.11, which assured that the drug is a promising candidate for commercial-scale production by direct compression as shown in Figure 1 and Supplementary Table S1. Moreover, the Parameter Indices (IP) value of the active substance was 0.83. The mean radius values for Cohesion Index (Icd) and Inter-Particle Porosity (Ie) were not in compliance with the target (≥5).

Similarly, the results of formulation blends (F1-F16) obtained from radar plots proved that each of the formulations had satisfactory compressional characteristics with an IGC value ≥ 5 as shown in Figure 1 and Supplementary Table S2.

### Pharmaceutical evaluation of core and coated tablets

#### Physical and chemical evaluation

The mean tablet weight was 501.51 ± 5.13 mg to 506.61 ± 7.96 mg, hardness was 9.84 ± 0.41 kg to 10.20 ± 0.43 kg, and the maximum percentage friability was 0.29%. The mean diameter and thickness of the core tablets were in the range of 11.00 ± 0.03 mm to 11.03 ± 0.05 mm and 5.50 ± 0.07 mm to 5.53 ± 0.07 mm, respectively (see Table 5). The results obtained from the content uniformity test of each formulation blend (*n = 20*) are illustrated in Table 5 (see Supplementary Figure S1–S4 for representative HPLC-UV chromatogram of blank (Supplementary Figure S1), placebo (Supplementary Figure S2), standard (Supplementary Figure S3), and sample (Supplementary Figure S4). All the batches met the official requirements of chemical assay limits (90–110%) (U.S.P.39-N.F.34, 2016).

**TABLE 5:** Physicochemical quality characteristics of tablets.

Type of tablets	Formulation code	Physical characteristics (*n* = 20)					Chemical characteristics (*n* = 20)
		Weight (mg)	Thickness (mm)	Diameter (mm)	Hardness (kg)	Friability (%)	Content uniformity (%)
Core tablets	F1-F4	502.34 ± 4.56	5.51 ± 0.07	11.01 ± 0.03	10.20 ± 0.43	0.18	97.6
	F5-F8	501.51 ± 5.13	5.50 ± 0.07	11.03 ± 0.05	10.08 ± 0.46	0.15	100.9
	F9-F12	504.83 ± 5.27	5.51 ± 0.06	11.00 ± 0.03	9.84 ± 0.41	0.29	99.3
	F13-F16	506.61 ± 7.96	5.53 ± 0.07	11.01 ± 0.03	10.06 ± 0.42	0.21	93.7
Coated tablets	F1-F2	543.51 ± 6.43	6.08 ± 0.07	11.57 ± 0.03	Not applicable		96.1
	F3-F4	564.91 ± 5.13	6.13 ± 0.07	11.67 ± 0.03			99.78
	F5-F6	543.72 ± 5.82	5.98 ± 0.07	11.50 ± 0.05			95.7
	F7-F8	564.83 ± 6.05	6.14 ± 0.09	11.61 ± 0.07			95.3
	F9-F10	544.72 ± 5.68	5.93 ± 0.10	11.53 ± 0.02			97.2
	F11-F12	566.44 ± 5.91	6.12 ± 0.07	11.67 ± 0.08			98.3
	F13-F14	550.25 ± 8.65	5.96 ± 0.08	11.47 ± 0.04			99.1
	F15-F16	566.71 ± 8.91	6.23 ± 0.04	11.65 ± 0.06			98.2

#### *In-vitro* drug release studies

**FIGURE 2:**
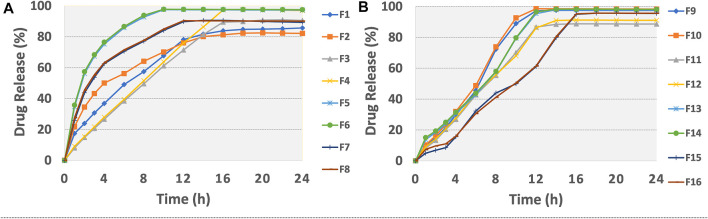
Release plots of tramadol HCl from formulations **(A)** F1-F8 and **(B)** F9-F16 in dissolution media 0.1 M HCl (pH 1.2) for 2 h and then in phosphate buffer (pH 6.8) from 3–24 h.

**FIGURE 3:**
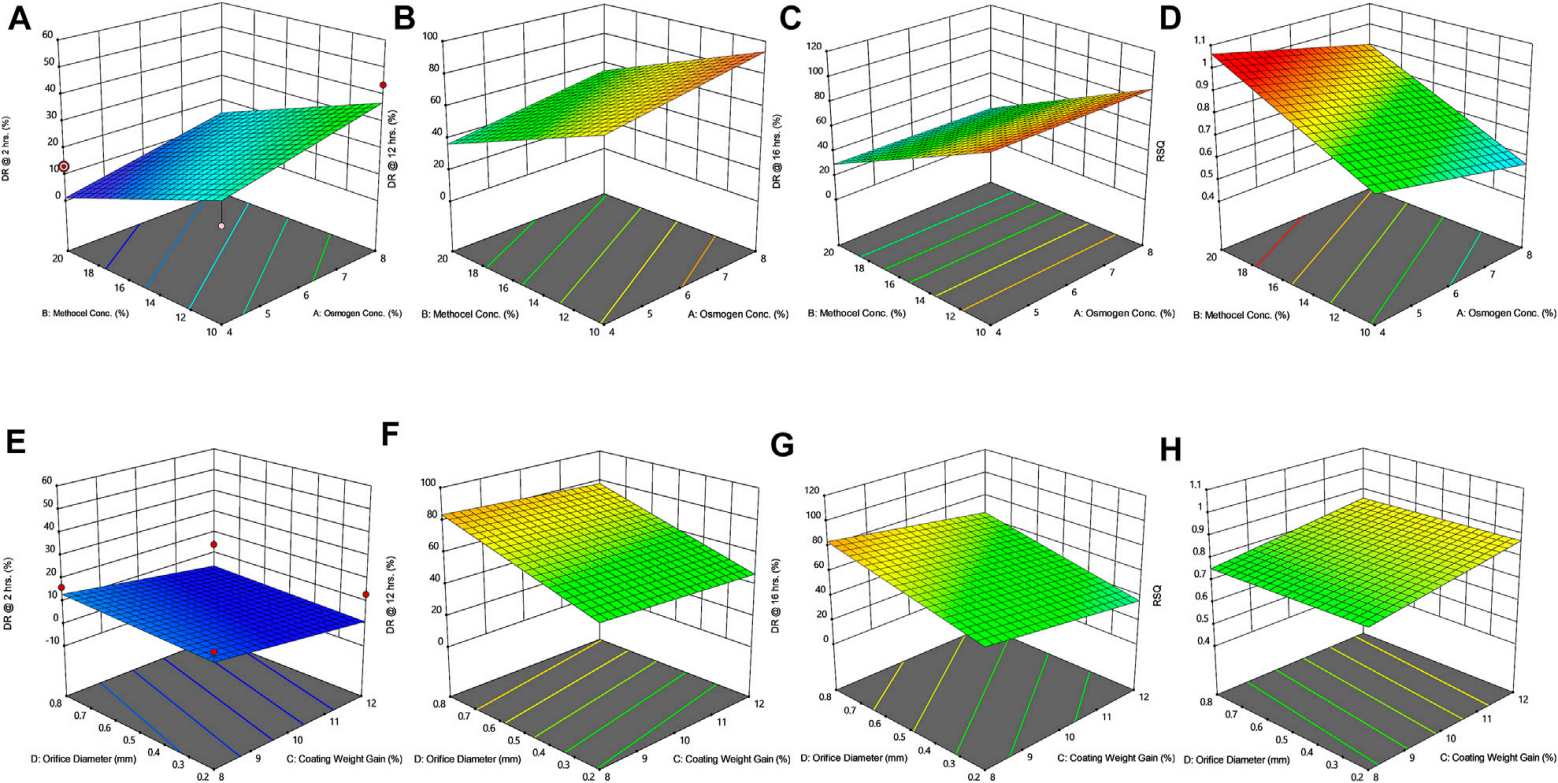
Response surface plots (3D) showing the effect of osmogen concentration (X_1_), Methocel™ K4M concentration (X_2_), coating weight gain (X_3_) and orifice diameter (X_4_) on the release of F3 formulation **(A)** at 2 h **(B)** at 12 h **(C)** at 16 h **(D)** on zero-order release coefficient (*r* ^2^).

**FIGURE 4:**
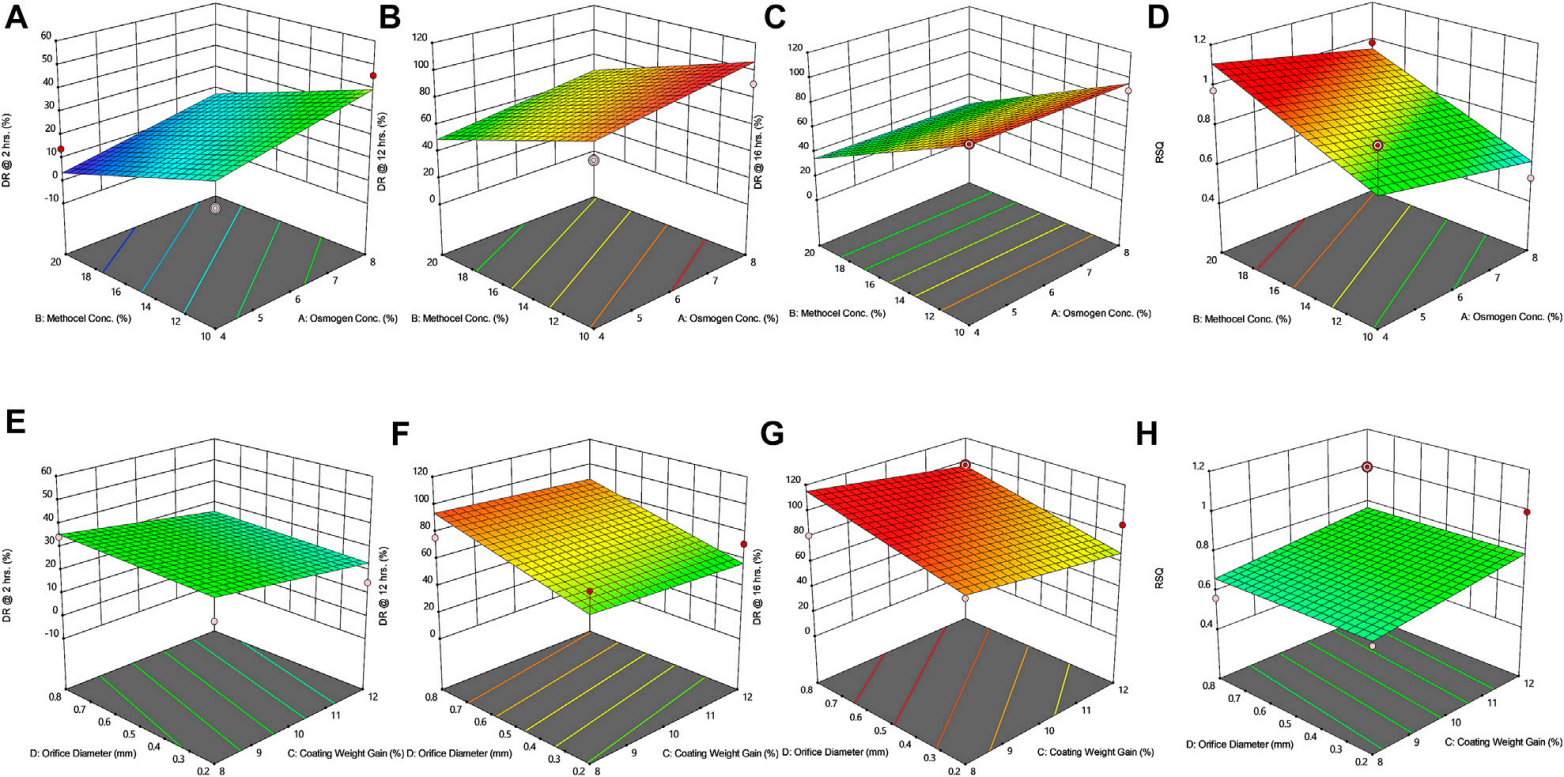
Response surface plots (3D) showing the effect of osmogen concentration (X_1_), Methocel™ K4M concentration (X_2_), coating weight gain (X_3_) and orifice diameter (X_4_) on the release of F4 formulation **(A)** at 2 h **(B)** at 12 h **(C)** at 16 h **(D)** on zero-order release coefficient (*r* ^2^).

The findings of *in vitro* drug release data are given in Table 6. The release profiles of different formulations are illustrated in Figures 2A,B. Table 7 presents model equations for drug release at 2 (Y1), 12 (Y2), and 16 h (Y3). The relative effect of concentration of osmogen and rate controlling polymer at various time points are expressed in Figures 3A–D, 4A–D. Similarly, the effect of percentage coating weight gain and orifice size is shown in Figures 3E–H, 4E–H.

**TABLE 6:** The release rate and coefficient of correlation of tramadol osmotic formulations calculated by Model Dependent Kinetics.

Formulations code	First order		Zero order		Higuchi		Korsmeyer-Peppas			Hixon-Crowell		Baker-lonsdale		Weibull		
	
	r^2^	k_1_	r^2^	k_o_	r^2^	k_H_	r^2^	n	k_KP_	r^2^	k_HC_	r^2^	k_BL_	r^2^	α	β
		*(hr* ^*−1*^ *)*		*(hr* ^*−1*^ *)*		*(hr* ^*−1/2*^ *)*			*(hr.* ^*-n*^ *)*		*(hr* ^*−1/3*^ *)*		*(hr* ^*−1*^ *)*			
F1	0.978	0.101	0.656	4.717	0.964	19.655	0.965	0.476	20.977	0.95	0.03	0.96	0.011	0.998	7.426	0.931
F2	0.929	0.139	0.562	6.423	0.98	22.03	0.993	0.42	26.371	0.98	0.033	0.943	0.011	0.993	3.844	0.675
F3	0.97	0.097	0.992	5.898	0.90	19.146	0.999	0.897	8.098	0.989	0.028	0.843	0.008	0.992	20.375	1.313
F4	0.962	0.097	0.994	6.271	0.959	20.308	0.997	0.902	8.02	0.977	0.031	0.826	0.009	0.984	22.609	1.407
F5	0.994	0.374	0.482	12.521	0.959	34.157	0.986	0.382	42.046	0.969	0.102	0.992	0.037	0.999	2.295	0.859
F6	0.994	0.389	0.457	12.651	0.954	34.561	0.986	0.373	42.213	0.971	0.106	0.993	0.039	0.999	2.217	0.86
F7	0.969	0.226	0.489	8.333	0.962	26.868	0.986	0.396	33.464	0.916	0.061	0.992	0.021	0.998	3.044	0.752
F8	0.964	0.238	0.522	9.419	0.971	28.069	0.99	0.402	33.982	0.907	0.064	0.994	0.022	0.997	2.857	0.734
F9	0.901	0.135	0.988	8.37	0.822	23.074	0.99	1.061	7.327	0.937	0.039	0.753	0.012	0.984	34.477	1.821
F11	0.946	0.111	0.989	6.81	0.863	20.499	0.992	0.951	7.643	0.972	0.032	0.802	0.009	0.991	25.13	1.501
F12	0.952	0.114	0.976	6.495	0.889	21.099	0.986	0.858	9.22	0.978	0.032	0.824	0.01	0.991	23.108	1.464
F13	0.92	0.129	0.985	7.498	0.87	22.644	0.988	0.929	8.856	0.953	0.037	0.798	0.012	0.967	23.425	1.553
F14	0.92	0.132	0.982	7.568	0.876	22.907	0.986	0.906	9.422	0.952	0.037	0.804	0.012	0.962	20.988	1.511
F15	0.894	0.081	0.978	5.475	0.78	17.266	0.992	1.229	3.095	0.925	0.024	0.723	0.006	0.979	77.28	1.806
F16	0.895	0.082	0.981	5.489	0.788	17.362	0.994	1.22	3.174	0.925	0.024	0.731	0.006	0.975	76.775	1.802

**TABLE 7:** Probability value of selected responses and regression coefficient of applied constraints.

Response	Y_1_	Y_2_	Y_3_	Y_4_
*p*-value	0.0021	0.0441	0.0419	0.0027
The regression coefficient of applied constraints				
Y_1_ = 25.258 + 6.871X_1_ - 11.159X_2_ - 4.846X_3_ + 1.267X_4_				
Y_2_ = 85.061 + 1.297X_1_ + 0.358X_2_ - 7.188X_3_ + 0.404X_4_				
Y_3_ = 93.201 + 2.209X_1_ + 2.124X_2_ - 0.948X_3_ + 0.629X_4_				
Y_4_ = 0.814–0.0795X_1_ + 0.169X_2_ 0.052X_3_ - 0.007X_4_				

#### Formulation optimization

The results of the targeted response (Y1-Y4) against different input variables (X1-X4) are explained as ramp plots in Supplementary Figures S5, S6. The results of two random described check point formulations with maximum desirability function (X1 = 2.11 and 2.74%, X2 = 14.09 and 12.48%, X3 = 8.73 and 11.92%, and X4 = 0.8 and 0.8 mm) were also assessed.

#### Effect of agitation rate and pH

**FIGURE 5:**
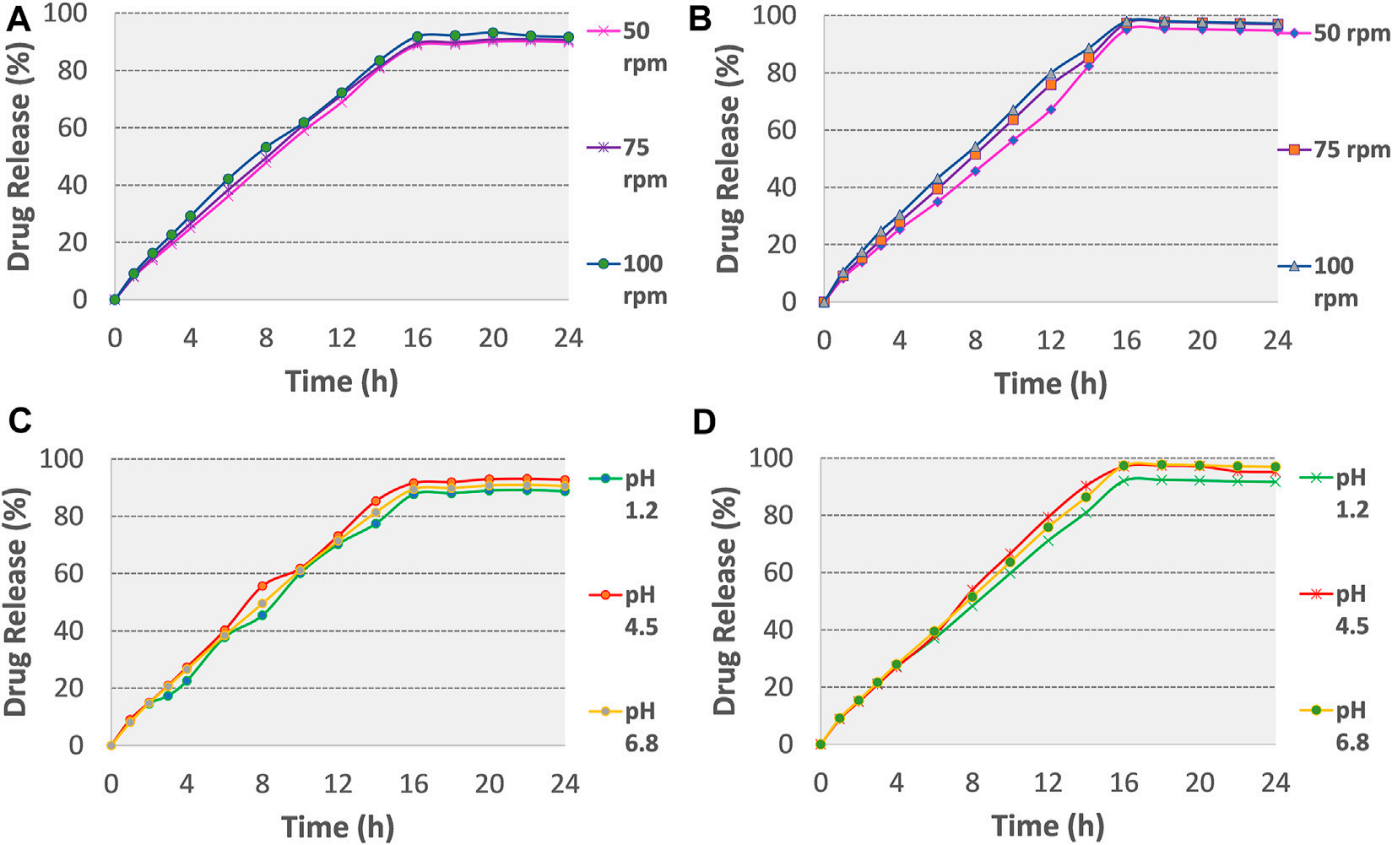
Release plots of tramadol HCl from formulation **(A)** F3 and **(B)** F4 in dissolution media 0.1 M HCl (pH 1.2) for 2 h and then in phosphate buffer (pH 6.8) from 3–24 h at variable stirring rates (50, 75, 100 rpm). Release plots of tramadol HCl from formulation **(C)** F3 and **(D)** F4 in various dissolution media with different pH values (pH 1.2, 4.5, 6.8; stirring rate 75 rpm).

The optimized formulations (F3 and F4) were examined for any change in the *in-vitro* release of the drug by varying the agitation speeds (50, 75, and 100 rpm) (see Figure 5). These optimized formulations were also subjected to *in-vitro* dissolution at three different pH conditions i.e., 0.1 N HCl pH 1.2, and phosphate buffers pH 4.5 and pH 6.8, as illustrated in Figures 5C,D and Table.

### Fourier transform infrared spectroscopy (FTIR) and scanning electron microscopy (SEM)

**FIGURE 6:**
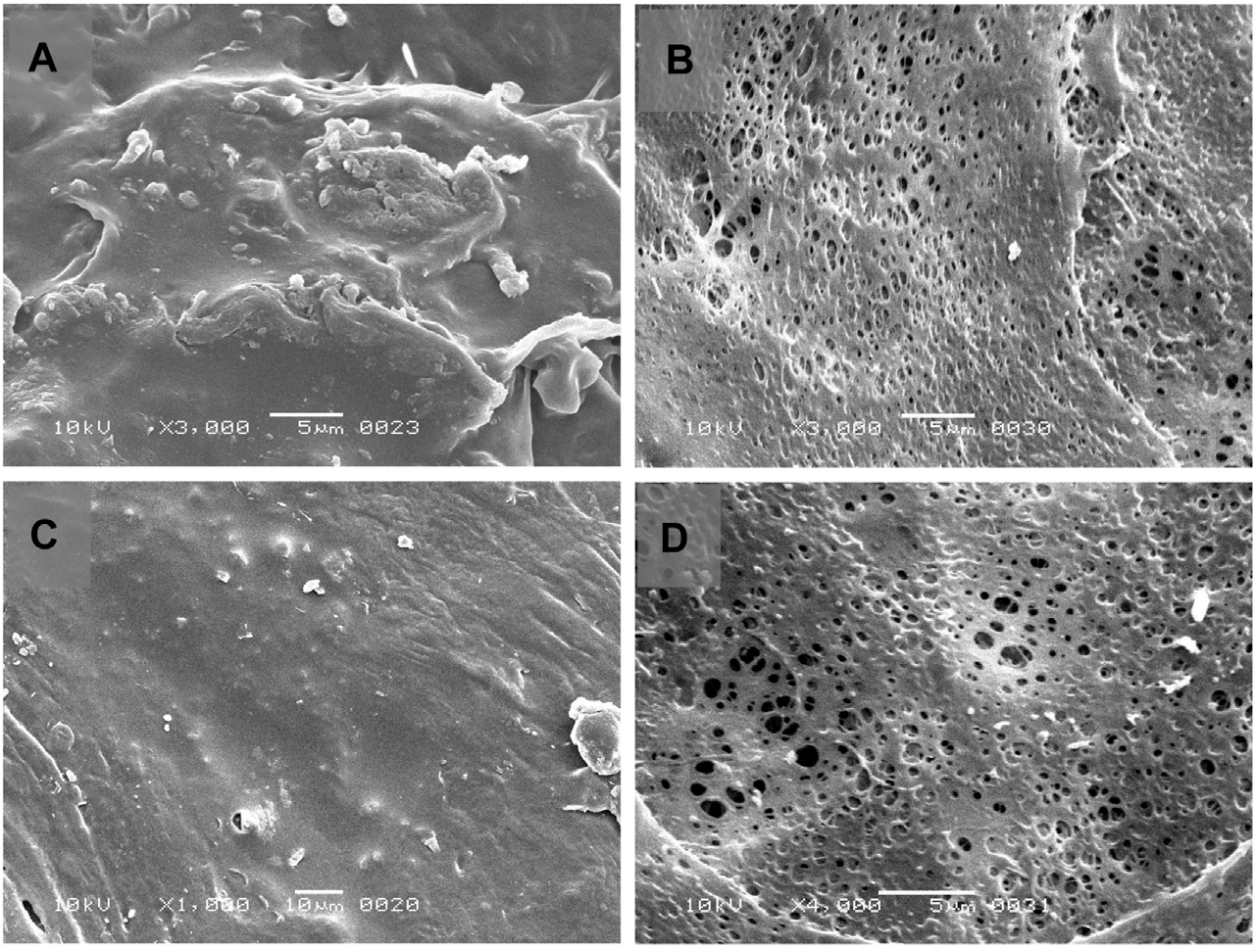
Scanning electron micrograph (SEM) of Opadry^®^ CA coating on core osmotic tablet formulations before dissolution **(A)** [F3] and **(C)** [F4] and after dissolution experiments **(B)** [F3] and **(D)** [F4].

The results obtained from FTIR peaks of the pure active drug along with the formulations F3 and F4 are illustrated in Supplementary Figures S7–S9. Similarly, the SEM images are expressed in Figure 6.

### Accelerated stability studies

The corresponding shelf lives of F3 and F4 were calculated using statistical software (Minitab version 17) and found 29.41 and 23.46 months, respectively.

### *In-silico* PBPK modeling and simulation

**FIGURE 7:**
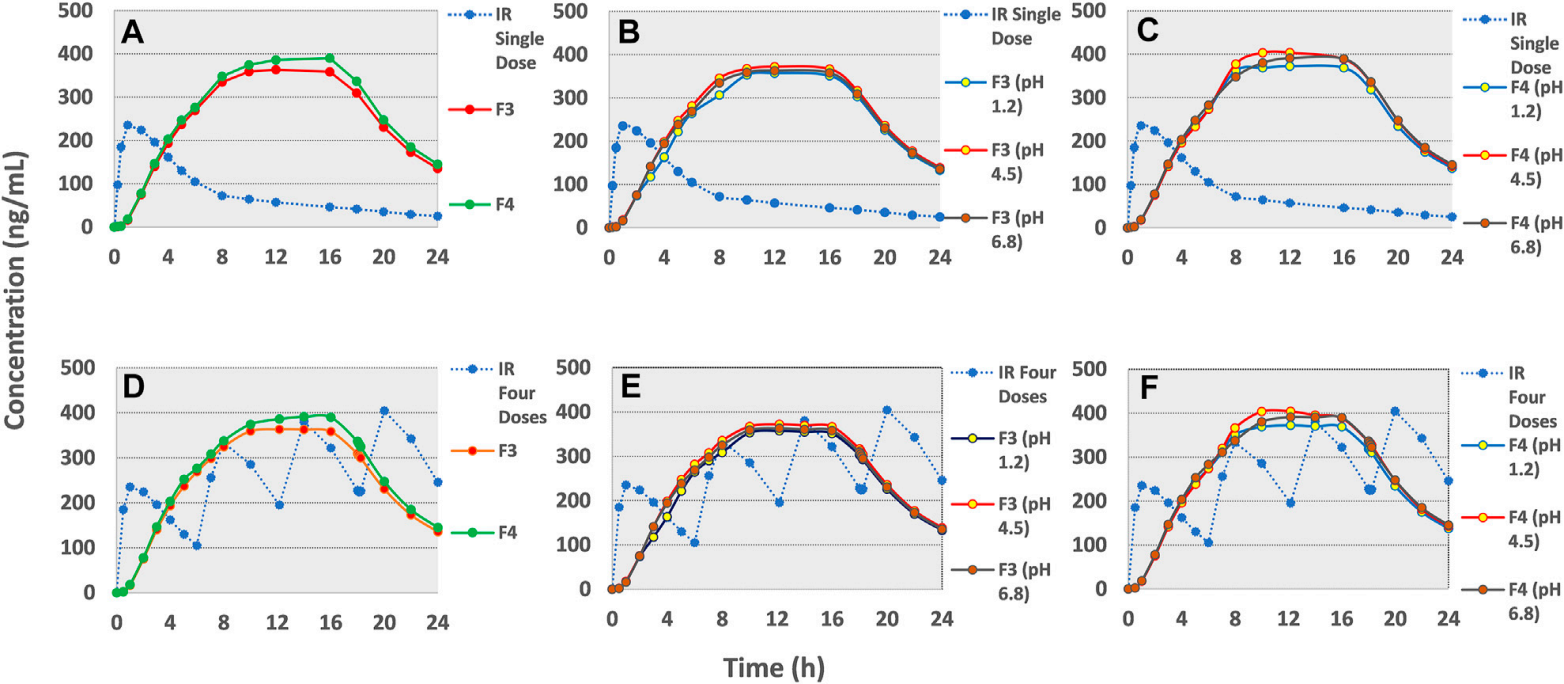
GastroPlus™ simulated drug plasma concentration-time profiles of **(A)**single dose IR tramadol HCl - 50 mg and once daily F3 and F4 tramadol- 190 mg osmotic tablets. Simulated profiles for effect of pH of dissolution media **(B)** on F3 and **(C)** on F4. Simulated profile of multiple dose (q6h for 24 h) IR tramadol HCl–50 mg and F3 and F4 **(D)** and in variable pH **(E)** for F3 and **(F)** F4.

The pharmacokinetic parametric values and plasma profiles for single and multiple doses of tramadol HCl 50 mg obtained from literature against the predicted plasma profiles of optimized formulations are shown in Table 8 and Figures 7A,D. Similarly, the plasma profiles of optimized formulations at different pH are also compared in Table 8 and Figures 7B,C,E,F.

**TABLE 8:** Calculated and predicted pharmacokinetic parameters of tramadol HCl formulations.

Pharmacokinetic parameters	Immediate release tramadol HCl - 50 mg^a^		Osmotic controlled-release (CR) tramadol HCl - 190 mg^b^							
	Single dose	Multiple dose (q6h for 24 h)	Once-daily F3 (simulated PK data)				Once-daily F4 (simulated PK data)			
	*in-vivo data* (parameters calculated with PKPlus™)		Drug release media (for ACAT model based *in-vivo* simulation)							
			2-stage^c^	pH 1.2	pH 4.5	pH 6.8	2-stage^c^	pH 1.2	pH 4.5	pH 6.8
*C*_*max*_ (ng/ml)	275.31	406.53	364.45	355.52	373.91	360.02	390.2	371.34	406.3	394.21
*T*_*max*_ (h)	1.12	20.74	11.28	10.87	13.15	12.33	14.97	14.2	11.8	14.14
*AUC*_*t*_ (ng/ml×h)	1524.9	6448.2	6176.2	5903.3	6383.7	6189.3	6441.9	6292.8	6515.5	6470.1
*AUC*_*inf*_ (ng/ml × h)	2164.1	7917.4	7388.4	7103.2	7559.6	7422.2	7663.8	7402.2	7827.8	7705.7

a Data acquired from Brvar et al. (2014) (9) and processed in PKPlus (Gastroplus™^,^ *ver* 9.8).

b The *in-vitro* release data in various dissolution media (0.1 N HCl pH 1.2, phosphate buffer pH 4.5 and 6.8) was incorporated in ‘ACT’ model in GastroPlus™^,^ for *in-vivo* pharmacokinetic simulation.

c 2-stage media comprised of 2 h in 0.1 N HCL pH 1.2 and then from 3–24 h in phosphate buffer pH 6.8 (8).

## Discussion

### Design of experiments (DoE) for elementary osmotic tablets

In the present work, an osmotically controlled tablet of tramadol HCl was developed for 24 h; this approach may reduce the requirement of repeated drug administration in a single day and eventually improve patient compliance, whereas, Kumar et al. *in* 2009 developed tramadol HCl osmotic tablets only for 12 h release (Kumar et al., 2009), which may need comparatively frequent drug administration for pain management. To obtain the desirable pharmaceutical parameters within the constraints, factorial design is the widely used DoE to generate sufficient data for formulation optimization based on a predictive model. Full factorial is a widely reported design of experiment methodology for osmotic tablets. In the current work, 16 formulation runs were obtained by applying two level four factors (2^4^) full factorial design. Habib et al., in 2014 used 2^3^ full factorial design to study the impact of different concentrations of rate controlling polymer and coating weight gain percentage on drug release and zero-order regression coefficient (Habib et al., 2014). Two-level factorial design is reported to be adequate when there is existing orthogonality between the dependent and independent variables (Khanvilkar et al., 2002; EL-Shorbagy et al., 2019; Omar et al., 2020). A linear relationship between percentage drug release and orifice diameter and weight gain after coating was also reported by Li et al. (Li Y. et al., 2019; Gundu et al., 2020). An extensive literature survey was performed to select process and formulation factors (Li N. et al., 2019; Cheng et al., 2020; Gioumouxouzis et al., 2020; Vrbanac et al., 2021). Vrbanac et al. also used Methocel™ K4M as rate controlling agent in sustained and controlled release oral dosage forms prepared by the direct compression method because of its good compressibility (Vrbanac and Krese, 2019). Sodium chloride was used as an osmogen, and it has been reported that even when used in smaller concentrations it produces high osmotic pressure, than any other osmogens (Liu et al., 2020; Nie et al., 2020). Nakajima et al., in 2018 studied the effect of different molecular weights of polyethylene oxide and variable orifice diameter on the dissolution profile of a drug from an elementary osmotic pump and reported a sigmoidal release profile with a reduction in orifice diameter (Nakajima et al., 2018). Gundu et al., in 2022 designed push-pull osmotic tablets of Ondensetrone, using five factors-two level full factorial design and studied the effect of different polymers and orifice diameters on drug release till 24 h (Gundu et al., 2020). Similarly, 3^2^ full factorial was also applied to observe the effect of coating weight gain on the release of vinpocetine from solid self-nano emulsifying (S-SNEDDS) asymmetrically coated osmotic tablets (El-Zahaby et al., 2018).

### Pre-formulation evaluation by SeDeM expert tool

For the evaluation of compression characteristics of pharmaceutical powder excipients and active pharmaceutical ingredients to form a tablet, the SeDeM expert tool was developed. In SeDeM-based assessment, twelve micromeritics parameters are linearized for the comparative evaluation of the physical characteristics of powders. This system has been used frequently for the pre-formulation assessment of immediate release dosage forms, for example, Gülbağ et al., in 2018 applied SeDeM as an expert tool to develop directly compressed memantine orally disintegrating tablets, using Ludiflash^®^, Ludipress^®^, and Parteck^®^ to improve compressibility of the drug (Gülbağ et al., 2018). In the current study for prolonged and effective pain management, controlled release tramadol osmotic tablet formulation was developed, and the SeDeM expert tool was applied for pre-compression evaluation of formulation ingredients. In designing an osmotic tablet, Methocel™ K4M was used as a vital rate-controlling polymer. The intrinsic IGC value of Methocel™ K4M was observed higher than that of Avicel PH-101^®^, similar findings were reported by Nardi-Ricart et al. that formulations containing a higher proportion of Methocel™ K4M exhibited better suitability for direct compression, especially when the compressibility of API (tolcapone) is poor (Nardi-Ricart et al., 2020).

The SeDeM diagrams revealed that powders and their blends bearing poor densities exhibited deficient parameters (*r*-value <5) as given in Figure 1. The minimum amount of Avicel PH-101^®^ required to rectify the lower value of deficient incidence factor (IF), for compressibility was calculated to be 37.04% so that the Cohesion Index (Icd) and Inter-Particle Porosity (Ie) of tramadol HCl get improved. Suñé-Negre et al. reported the Icd and Ie values of Avicel PH-101^®^ as 10.00 and 6.02, respectively, which are closer to the current findings (see Supplementary Table S1) (Suñé-Negre et al., 2008).

### Compression of core tablet and formation of semipermeable membrane

The core tablets of trial formulations (F1-F16) containing 190 mg of tramadol HCl were compressed directly, then subjected to coating by Opadry^®^ CA dispersion as the semipermeable membrane former. Farooqi et al., in 2020 also applied Opadry^®^ CA as a semipermeable membrane former in developing an osmotically controlled release metoclopramide HCl tablet (Farooqi et al., 2020). The uniform stretching of the coating polymer onto the surface of the core tablet is shown in SEM images alongwith the presence of an evident orifice. Different researchers have created the orifice size of 0.2–1mm, to study the effect of orifice diameter on controlling drug release (Shokri et al., 2008).

### Pharmaceutical evaluation of core and coated tablets

#### Physical and chemical evaluation

The weight variation of all the trials (F1-F16) was found within the limit of ±5% as prescribed by USP (U.S.P.38-N.F.33, 2014). The physical strength of core tablets to withstand the mechanical shock, especially during the coating process, was also found satisfactory. Uniformity of thickness and diameter is also essential for making a firm coating layer and providing a smooth surface area for uniform water uptake. Yang et al. applied an electrostatic powder coating process for the uniform deposition of coating polymer cellulose acetate on the surface of core tablets (Yang et al., 2020). Finally, the results of content uniformity and assay suggest that all the formulation blends fall in the acceptable ranges and thus satisfy physicochemical evaluation.

#### *In-vitro* drug release studies

The drug release profile of tramadol HCl showed that formulations F1, F2, and F5-F8 largely followed first order release kinetics with the variable initial burst release. The maximum release (∼80–90%) from these formulations was up to 10–12 h except for F6, which exhibited a maximum release of >95% within 10 h (see Figure 2A). On the contrary, formulations (F9-F16) containing a high level (+1) of Methocel™ K4M released tramadol HCl at a zero-order rate, but the erratic release behavior was observed (see Table 6 and Figure 2B), which may be possibly associated with the excessive swelling of some units within 6–12 h. Lin et al. also reported that when the outer coating is non-extensible and could not uphold a high intrinsic osmotic pressure, the osmotic pump suffered a failure which may lead to burst release (Lin et al., 2002). Only two formulations, F3 and F4, followed zero-order release rates, Y4 (0.992 and 0.994) which was observed independent of orifice size (F3 = 0.2 mm, F4 = 0.8 mm), however, Farooqi et al. reported that initial burst release from osmotic tablets was due to large orifice size and minimal weight gain after coating (Farooqi et al., 2020). Moreover, in the present work, the maximum release was found to be more than 90%, up to 16 h. This shows that the inclusion of matrix forming polymer effectively controlled the tramadol HCl release rate, especially in the initial hours; however, at the 12th hour, the effect started to decrease (see Figures 3A–D, 4A–D).

The positive coefficient values for Y1-Y3 and 3D surface curves (Figures 3A–D, 4A–D) also exhibited that the percentage of drug release increased with the rise in osmogen (sodium chloride) concentration. However, this effect was also found to be at initial hours, whereas at 12 and 16 h time points, only a marginal effect was observed. The possible reason for this release behavior may be due to a very high osmotic pressure (356 atm) produced by sodium chloride saturated solution, which promotes the drug content release from the tablet core. It has been previously reported that the release rate of drugs from the elementary osmotic tablet increases with the increment of the osmotic agent in the formulation (Ning et al., 2011; Ali and Sayed, 2013).

The coating thickness was ascertained by determining the weight gain during the coating process of Opadry^®^ CA. As expected, the higher weight gain of 12% (+1) led to a significant decrease in the drug release compared to the lower weight gain of 8% (−1). This may be explained in a way that a higher coating gain would eventually slow the entry of external fluid inside the core. This would result in the development of lesser osmotic pressure inside the core, thereby promoting a lesser drug efflux (see Figures 3E–H, 4E–H). A study has previously reported that a higher coating weight reduces the release of atenolol (Arjun et al., 2016). Similar results were also found in a study that showed that the release rate is inversely linked with the increment of coating gain (Özdemir and Sahin, 1997). However, this phenomenon was not observed at 16 h, possibly since most of the drug was already released by the time from the system, and there was lesser osmotic pressure on the tablet to push the drug out of the orifice. The coefficient values in the model equation of each term are also in agreement with this effect (Table 7).

Similarly, very small values of coefficients were observed for RSQ (Y4) in model equations (Table 7), showing the lack of impact of orifice size on zero order release rate (see Figures 3E–H, 4E–H). This could be because of the high aqueous solubility and dissolution rate of the drug, which tend to mask the role of the orifice opening (0.2–0.8 mm) on drug release from osmotic tablets. However, the interactive combined role of Osmogen (X1), Methocel™ K4M (X2), and tablet weight gain after coating (X3) with Opadry^®^ CA, was observed enough to exert control over the release of the drug for 16 h. It has been reported previously by Liu L et al. that the release of the highly soluble drug, nifedipine HCl, from the monolithic osmotic system was unaffected by the change in orifice size (Liu et al., 2003). Similarly, a very minimal effect was observed for the release of Captopril by varying the size of the opening in an elementary osmotic pump (Xu et al., 2003).

The results obtained after applying drug-release kinetic models suggest that the formulations (F9-F16) with a higher proportion of matrix-forming polymer (+1) follow the zero-order release profile (*r* ^2^ = 0.991–0.976) as well as the Korsmeyer-Peppas kinetics (*r* ^*2*^ = 0.994–0.986) (see Table 5). However, a lower proportion of Methocel™ K4M (-1) in many formulations could not produce sufficient control on the release. Thus, the first-order release is pre-dominant except for F3 and F4, which fitted best to zero-order release rates. The values of the Korsmeyer-Peppas rate constant (*k*_*KP*_), correlation coefficient (*r* ^*2*^), and release exponent (*n*) of all the developed formulations are also presented in Table 6. The findings suggested that at a lower level (-1) of X2, the system preferably follows the *Fickian diffusion,* as observed for F1, F2, and F5-8 with *n* value ≤0.5. This effect is reported to be associated with the phase transformation of polymeric chains into a glassy state; thereby, the rapid release of dissolved drug molecules ensues. On the contrary, with “*n*” value >0.85 for F9-F12 indicated “*case II*” transport with nearly zero-order release (Siepmann and Peppas, 2012). This *case II* effect may be due to swelling and bursting effects of Methocel™ K4M and consequently stress-induced (osmotic pressure) deformations of glassy polymeric chains at the interface of the non-distensible semipermeable membrane created by Opadry^®^ CA (Thomas and Windle, 1982). Similarly, “*super case-II’*”transport (“*n’*”values >1), was observed for F15 and F16, which was likely due to a higher level (+1) of polymer (X2) and osmogen (Xl). The study by Khan et al. also advocated that by changing formulation variables, the value of release exponent (*n*) may reach up to 1.45 (Khan et al., 2011). For formulation F3 and F4, the “*case II*” transport phenomenon was observed “*n*” values of around 0.9 (>0.85; *case II* zero-order). The reason could be attributed to higher coating weight gain, which led to the decreased influx of water through the membrane, and a consequently lesser rate of solubilization of the drug compared to formulations with a lower level of coating. Secondly, the formation of a non-distensible membrane by coating polymer may have exerted enough pressure to cause the deformation of a glassy chain of swelled polymer and consequently change the release kinetics from “*Fickian*” to “*case II*” transport was observed (Meares et al., 1977; Fu and Kao, 2010).

#### Formulation optimization

Based on a multi-criteria approach the finest quantities of input variables (X1-X4) were optimized to achieve the targeted responses (Y1-Y4). The desirability function of numerical optimization was applied for, which constraints were set as given in Table 1, and ramp plots are best defined the optimum values of X1-X4 for the targeted responses (Supplementary Figures S5, S6). Two random checkpoint formulations (I and II) with maximum desirability values were selected to validate the experimental model. The results of checkpoint formulations on response variables (Y1-Y3) values were observed in close agreement with the formulation F3 and F4 values of the DoE. However, the RSQ_zero_ (Y4) of F3 and F4 were observed to be slightly greater than that of checkpoint formulations I and II (F3 = 0.992 and F4 = 0.994, I = 0.900, II = 0.902). Gong et al., in 2018 also used numerical optimization technique to generate optimum amounts of variables and compared the predicted and observed values of each critical output variable to validate the full factorial experimental design for gastric floating sustained release mini tablets of alfuzosin HCl (Gong et al., 2018).

#### Effect of agitation rate and pH on drug release

No change was observed in the mean drug release of optimized formulation F3 and F4 till 16 h (see Figures 5A,B and Supplementary Table S3), this showed that gastrointestinal motility would not influence the release of tramadol HCl from osmotic tablets. In a reported study, Lu et al. studied the effect of variable stirring rate (50, 100, and 150 rpm) and pH (1.2, 5, and 6.8) on the dissolution profile and observed no significant effect on the release rate of naproxen from monolithic osmotic tablets (Lu et al., 2003). In the present study, Figures 5C,D and Supplementary Table S3 also demonstrated that the release of tramadol HCl from the osmotic pump is pH-independent. The closest comparison of dissolution data at different pH and agitation rates using the *f*_2_ similarity factor test showed similar release profiles as shown in Supplementary Table S4. The possible reported reason is the outer coating of Opadry^®^ CA, which is impermeable for the movements of ions, thus excluding the effects of surrounding fluids on the tablet core (Theeuwes, 1975; Wang et al., 2008).

### Fourier transform infrared spectroscopy (FTIR) and scanning electron microscopy (SEM)

The spectra obtained from FTIR are indicative of the presence of these functional groups: aromatic C = C stretching (1580–1610 cm^−1^), -OH stretching and bending (3300–3310 cm^−1^), -CH stretching (2920–2930 cm^−1^), asymmetric carbon-carbon double bond stretching (1920–1940 cm^−1^), C-C stretching (825 cm^−1^), CH_2_ rocking (1415 cm^−1^) and -CH_3_ bending vibrations (1479 cm^−1^). The scans of formulations spectra were similar to that of pure tramadol HCl and assured no observable interaction with formulation ingredients. A study conducted by Palla N et al. also reported the absence of interaction between tramadol HCl and HPMC (Palla et al., 2013). Another study suggests that there is no visible interaction between tramadol HCl and commonly used pharmaceutical excipients (Flores-Arriaga et al., 2021).

### Scanning electron microscopy (SEM)

The SEM visualization (see Figure 6) of coated tablets before dissolution showed the presence of a plain and uniform coating layer of Opadry^®^ CA without having any pores or cracks on both the optimized formulations F3 and F4, and the morphological composition of tablets remained intact. In contrast, after dissolution (and overnight drying at 50°C), numerous micropores were developed on the surface of the tablets. This phenomenon is well documented in the literature, Verma et al. observed that the percentage porosity increased with the increasing concentration (0–55%) of pore former (polyvinyl pyrrolidone) and produced the anticipated effect on the percentage drug release, whereby micropores appear in the coating layer after contact with dissolution media (Verma et al., 2003).

### Accelerated stability studies

Following the ICH guidelines (International Council for Harmonization, 2003), the optimized formulations were tested for stability studies under accelerated conditions of 40 ± 3°C and 75 ± 5% relative humidity for up to 6 months. These results manifested, that the optimized formulations F3 and F4 remained stable over time without changes in physical parameters, content uniformity, and release profiles (Supplementary Table S5). Ahmed et al. also observed insignificant changes in the physical appearance and dissolution profile of the osmotic delivery system of eprerisone HCl (Ahmed et al., 2018).

### *In-silico* PBPK modeling and simulation

The pharmacokinetic parameter estimation (PKPlus) of the *in-vivo* plasma drug concentration data reported by Brvar et al. (2014) revealed that a single dose of tramadol HCl 50 mg achieved an *AUC*_*inf*_ value of 2164.1 ng/ml×h with *C*_max_ of 275.3 ng/ml and *T*_max_ of 1.12 h (post-administration (see Table 8 and Figure 7A). Upon projecting this data for multiple-dose administration (50 mg q6h; 4 times), the “*C*_max_”, “*AUC*_*t*_” and “*AUC*_*inf*_” values were estimated as 406.53, 6448.2, and 7917.4 ng/ml × h respectively (see Table 8 and Figure 7D). After incorporation of various physicochemical, physiological, and obtained pharmacokinetic data of tramadol HCl in the “Absorption and Continuous Transit” (ACAT) model of Gastroplus™ *ver* 9.8 (see Table 3), the simulated plasma drug concentration-time profiles of the optimized formulations F3 and F4 showed a gradual and almost zero-order increase of plasma concentration at almost 4–5 h post-administration followed by *C*_max_ at about ∼10 h (F3) and 8 h (F4) (see Figure 7 (a)). This concentration was almost maintained for another 10 h (up to ∼16 h) and eventually followed by a first-order decline phase. The mean *C*_max_ values for optimized formulations F3 and F4 were calculated as 364.4 and 390.2 ng/ml. The *AUC*_*t*_ was 6176.2 and 6441.9 ng/ml × h and the *AUC*_*inf*_ was 7388.4 and 7663.8 ng/ml × h (see Table 8). For tramadol dose range of 50–400 mg the plasma concentration and area under the concentration-time curve relation is linear (Grond and Sablotzki, 2004). The toxic plasma concentration of tramadol is reported to be 2 mg/L (2000 ng/ml) (Pothiawala and Ponampalam, 2011) and according to another reported study, the mean plasma concentration of tramadol in 60 subjects with seizures was 491.90 µg/ml (Mohammadpour et al., 2019). The current study finding revealed that the maximum plasma concentrations simulated by *in-silico* PBPK modeling for these optimized formulations F3 and F4 are far lesser than the reported toxic concentrations of tramadol.

Since it has been demonstrated in the preceding section that the pH of the release medium had a very negligible role in modifying the release of the drug, therefore, it didn’t translate significantly towards the *in-vivo* (simulated) profile as well (see Table 8 and Figure 7B,C,E,F).

After comparing it with the multiple dosed IR tramadol formulation, the simulated pharmacokinetic profiles of the optimized formulations (in 2-stage drug release medium; 2 h in pH 1.2 and 3–24 h in pH 6.8) were found to have very close “Relative Bioavailability” (F3, 98.23%; F4, 101.9%) and *C*_max_ values (F3, 364.4 vs. 406.53 ng/ml; F4, 390.2 vs. 406.53 ng/ml) (see Table 8 and Figure 7D. The “Relative Bioavailability” of the simulated profiles estimated from the drug release media with different pH values were also found to be well within the acceptable range of 80–125% (F3–pH 1.2, 94.4%, pH 4.5, 100.5%, pH 6.8, 98.6%; F4–pH 1.2, 98.4%, pH 4.5, 104.0, pH 6.8, 102.5%) (Chen et al., 2001; Davit et al., 2016). This validates that these osmotic controlled-release tramadol HCl formulations (F3 and F4) are sufficient for maintaining adequate plasma drug concentration profiles for once-daily dosing (U.S.P.40-N.F.35, 2016).

## Conclusion

Based on these findings, the SeDeM tool diagram is an expert way to design tramadol HCl, core tablets by direct compression method. Elementary osmotic pump technology using 2^4^ factorial design was successfully applied to develop once-daily tramadol HCl controlled-release tablets with Opadry^®^ CA for the robust delivery of highly aqueous soluble drug at zero-order rate. *In-vitro* investigation revealed that the system behaved independently of external influences of pH and GI motility. The *in-silico* PBPK-based modeling and simulations for *in-vivo* pharmacokinetics using GastroPlus™ (F3 and F4) strongly suggested that the release profiles in dissolution media (pH 1.2, 4.5, and 6.8) exhibited a good prediction for *in-vivo* performance. Moreover, the simulated *‘AUC*_*inf*_ *’* values were comparable with cumulative *‘AUC*_*inf*_ *’* from the simulated four multiple doses of the tramadol HCl 50 mg (6 hourly dosings) thereby supporting the case of optimized formulations as sufficient for once-daily dosing.

## Data Availability

The original contributions presented in the study are included in the article/Supplementary Material, further inquiries can be directed to the corresponding author.
