# Supplementary material for: SeDeM tool-driven full factorial design for osmotic drug delivery of tramadol HCl: Formulation development, physicochemical evaluation, and in-silico PBPK modeling for predictive pharmacokinetic evaluation using GastroPlus™
Source: Front Pharmacol. 2022 Oct 7;13:974715. doi: 10.3389/fphar.2022.974715 (PMC9585207; doi:10.3389/fphar.2022.974715)
Supplement: Supplementary file 1 [file DataSheet1.docx]

**Supplementary**

**Table S1. SeDeM parameter experimental values of Tramadol HCl, Avicel pH 101®, and Methocel^TM^ K4M**

| **S. No.** | ***Parameter/ Indices*** | ***SeDeM experimental values for API and Excipients*** | | |
| --- | --- | --- | --- | --- |
|  |  | **Tramadol** | **Avicel PH 101^®^** | **Methocel^TM^ K4M** |
|  | *Parameter* |  |  |  |
| 1 | Bulk Density (Da) | 5.73 | 3.39 | 5.92 |
| 2 | Tapped Density (Dc) | 7.66 | 4.47 | 9.41 |
| 3 | Inter-particle Porosity (Ie) | 3.66 | 6.12 | 5.23 |
| 4 | Carr's Index (IC) | 5.04 | 5.01 | 7.43 |
| 5 | Cohesion Index (Icd) | 2.70 | 10.00 | 10.00 |
| 6 | Hausner’s Ratio (IH) | 8.32 | 5.55 | 7.05 |
| 7 | Angle of Repose (α) | 5.47 | 3.46 | 1.90 |
| 8 | Powder Flow (t") | 6.66 | 0 | 0 |
| 9 | Loss on Drying (%HR) | 6.95 | 3.84 | 3.97 |
| 10 | Hygroscopicity (%H) | 9.12 | 8.17 | 7.87 |
| 11 | Particles <50 µm (%Pf) | 9.92 | 3.38 | 0.00 |
| 12 | Homogeneity Index (IƟ) | 5.75 | 10.00 | 1.94 |
|  |  |  |  |  |
|  | *Mean Incidence Factor* |  |  |  |
| 1 | Dimension | 6.69 | 3.93 | 7.66 |
| 2 | Compressibility | 3.80 | 7.04 | 7.55 |
| 3 | Flowability/ Powder Flow | 6.82 | 3.00 | 2.98 |
| 4 | Lubricity/ Stability | 8.03 | 6.01 | 5.92 |
| 5 | Lubricity/ Storage | 7.84 | 6.69 | 0.97 |
|  |  |  |  |  |
|  | *Indices* |  |  |  |
| 1 | Parameter Index (IP) | 0.83 | 0.5 | 0.67 |
| 2 | Parameter Profile Index (IPP) | 6.41 | 5.28 | 5.06 |
| 3 | Reliability Factor | 0.952 | 0.952 | 0.952 |
| **4** | **Good Compressibility Index (IGC)** | **6.11** | **5.03** | **5.46** |
|  |  |  |  |  |
|  | Remarks* | A | A | A |
|  | * A = Acceptable *N/A = Not Acceptable | | | |

**Table S2. SeDeM parameters experimental values of different formulation blends of osmotic tablets of Tramadol HCl**

| **S. No.** | ***Parameter/ Indices*** | ***SeDeM experimental values for Formulations Blends*** | | | |
| --- | --- | --- | --- | --- | --- |
|  |  | **F1-F4** | **F5-F8** | **F9-F12** | **F13-F16** |
|  | *Parameter* |  |  |  |  |
| 1 | Bulk Density (Da) | 4.29 | 4.27 | 4.89 | 4.86 |
| 2 | Tapped Density (Dc) | 5.86 | 5.79 | 6.80 | 6.76 |
| 3 | Inter-particle Porosity (Ie) | 4.67 | 4.61 | 5.19 | 5.14 |
| 4 | Carr's Index (IC) | 4.91 | 4.83 | 5.65 | 5.59 |
| 5 | Cohesion Index (Icd) | 6.52 | 6.47 | 7.52 | 7.45 |
| 6 | Hausner’s Ratio (IH) | 6.36 | 6.31 | 7.07 | 7.01 |
| 7 | Angle of Repose (α) | 3.83 | 3.77 | 4.02 | 3.97 |
| 8 | Powder Flow (t") | 2.53 | 2.48 | 2.53 | 2.48 |
| 9 | Loss on Drying (%HR) | 4.77 | 4.71 | 5.16 | 5.11 |
| 10 | Hygroscopicity (%H) | 7.93 | 7.88 | 8.71 | 8.63 |
| 11 | Particles <50 µm (%Pf) | 5.29 | 5.23 | 5.29 | 5.25 |
| 12 | Homogeneity Index (IƟ) | 7.69 | 7.66 | 8.69 | 8.61 |
|  |  |  |  |  |  |
|  | *Mean Incidence Factor* |  |  |  |  |
| 1 | Dimension | 5.08 | 5.03 | 5.84 | 5.81 |
| 2 | Compressibility | 5.37 | 5.30 | 6.12 | 6.06 |
| 3 | Flowability/ Powder Flow | 4.24 | 4.19 | 4.54 | 4.49 |
| 4 | Lubricity/ Stability | 6.35 | 6.30 | 6.94 | 6.87 |
| 5 | Lubricity/ Storage | 6.49 | 6.45 | 6.99 | 6.93 |
|  |  |  |  |  |  |
|  | *Indices* |  |  |  |  |
| 1 | Parameter Index (IP) | 0.58 | 0.67 | 0.75 | 0.75 |
| 2 | Parameter Profile Index (IPP) | 5.39 | 5.33 | 5.96 | 5.91 |
| 3 | Reliability Factor | 0.952 | 0.952 | 0.952 | 0.952 |
| **4** | **Good Compressibility Index (IGC)** | **5.13** | **5.5** | **5.67** | **6.61** |
|  |  |  |  |  |  |
|  | Remarks* | A | A | A | A |
|  | * A = Acceptable *N/A = Not Acceptable | | | | |

**Table S3. In-vitro Release profiles of F3 and F4 at different pH and Agitation Rate**

| **Time (hr.)** |  | **F3-Mean % Drug Released (n=6)** | | | | | |  | **F4-Mean % Drug Released (n=6)** | | | | | |
| --- | --- | --- | --- | --- | --- | --- | --- | --- | --- | --- | --- | --- | --- | --- |
|  |  | **pH 1.2** | **pH 4.5** | **pH 6.8** | **50 rpm** | **75 rpm** | **100 rpm** |  | **pH 1.2** | **pH 4.5** | **pH 6.8** | **50 rpm** | **75 rpm** | **100 rpm** |
| 0 |  | 0 | 0 | 0 | 0 | 0 | 0 |  | 0 | 0 | 0 | 0 | 0 | 0 |
| 1 |  | 8.23 | 9.04 | 8.03 | 8.01 | 8.03 | 9.15 |  | 8.88 | 8.79 | 9.11 | 8.28 | 9.11 | 10.43 |
| 2 |  | 14.54 | 14.96 | 14.79 | 13.87 | 14.79 | 16.25 |  | 14.97 | 14.82 | 15.36 | 13.96 | 15.36 | 17.59 |
| 3 |  | 17.34 | 20.9 | 20.66 | 19.37 | 20.66 | 22.72 |  | 21.11 | 20.91 | 21.67 | 19.7 | 21.67 | 24.81 |
| 4 |  | 22.54 | 27.34 | 26.61 | 24.95 | 26.61 | 29.24 |  | 27.21 | 26.95 | 27.93 | 25.39 | 27.93 | 30.56 |
| 6 |  | 37.74 | 40.23 | 38.39 | 36.32 | 38.39 | 42.18 |  | 37.01 | 38.08 | 39.46 | 34.96 | 39.46 | 43.18 |
| 8 |  | 45.42 | 55.65 | 49.56 | 47.85 | 49.56 | 53.23 |  | 48.34 | 53.92 | 51.55 | 45.67 | 51.55 | 54.31 |
| 10 |  | 60.09 | 61.84 | 61.12 | 59.01 | 61.12 | 61.88 |  | 59.76 | 66.65 | 63.72 | 56.45 | 63.72 | 67.12 |
| 12 |  | 70.12 | 73.12 | 71.41 | 68.94 | 71.41 | 72.32 |  | 71.15 | 79.36 | 75.87 | 67.22 | 75.87 | 79.92 |
| 14 |  | 77.45 | 85.32 | 81.38 | 80.75 | 81.38 | 83.55 |  | 80.97 | 90.31 | 86.34 | 82.37 | 85.34 | 88.67 |
| 16 |  | 87.68 | 91.62 | 89.45 | 88.76 | 89.45 | 91.84 |  | 92.04 | 97.02 | 97.34 | 95.05 | 97.34 | 97.96 |
| 20 |  | 88.96 | 92.94 | 90.76 | 90.06 | 90.76 | 93.18 |  | 92.15 | 97.13 | 97.45 | 95.16 | 97.45 | 97.68 |
| 22 |  | 89.12 | 93.14 | 90.92 | 90.22 | 90.92 | 92.06 |  | 91.89 | 95.35 | 97.18 | 94.89 | 97.18 | 97.41 |
| 24 |  | 88.76 | 92.72 | 90.55 | 89.85 | 90.55 | 91.68 |  | 91.68 | 95.13 | 96.95 | 94.67 | 96.95 | 97.21 |

**Table S4. Similarity Factor (*f_2_*) determination of Tramadol HCl Osmotic Pump Tablets**

| **Comparison** | ***‘f_2_’* with reference to F3 in pH 6.8** | **Dissolution Profile** |
| --- | --- | --- |
| F3 in pH 1.2 | 88.59 | Similar |
| F3 in pH 4.5 | 91.03 | Similar |
| **Comparison** | ***‘f_2_’* with reference to F3 at 75 rpm** | **Dissolution Profile** |
| F3 at 50 rpm | 92.34 | Similar |
| F3 at 100 rpm | 86.55 | Similar |
| **Comparison** | ***‘f_2_’* with reference to F4 in pH 6.8** | **Dissolution Profile** |
| F4 in pH 1.2 | 89.29 | Similar |
| F4 in pH 4.5 | 89.76 | Similar |
| **Comparison** | ***‘f_2_’* with reference to F4 at 75 rpm** | **Dissolution Profile** |
| F4 at 50 rpm | 88.38 | Similar |
| F4 at 100 rpm | 86.02 | Similar |

**Table S5. Stability Data and Shelf Life of F3 and F4 at Accelerated Stability Conditions (40 ± 2% °C & 75 ± 5% RH)**

| **Study Duration** | **Physical Appearance** | **Drug Content (%)** | | | **Dissolution Change** | **Shelf Life (Months)** |
| --- | --- | --- | --- | --- | --- | --- |
|  |  | **Sample 1** | **Sample 2** | **Sample 3** |  |  |
|  |  | **n=20** | | |  |  |
| **Formulation containing 4% NaCl, 10% Methocel^TM^ K4M, 12% Weight Gain and Orifice Diameter of 0.2 mm (F3)** | | | | | | |
| 0 Month |  | 97.78 | 98.53 | 96.66 |  | **29.41** |
| 1 Month | Unchanged | 97.16 | 98.21 | 96.33 | Negligible |  |
| 3 Months | Unchanged | 96.58 | 97.49 | 95.92 | Negligible |  |
| 6 Months | Unchanged | 96.03 | 96.87 | 95.21 | Negligible |  |
| **Formulation containing 4% NaCl, 10% Methocel^TM^ K4M, 12% Weight Gain and Orifice Diameter of 0.8 mm (F4)** | | | | | | |
| 0 Month |  | 96.54 | 98.56 | 98.22 |  | **23.46** |
| 1 Month | Unchanged | 95.83 | 97.73 | 97.51 | Negligible |  |
| 3 Months | Unchanged | 94.99 | 97.12 | 96.98 | Negligible |  |
| 6 Months | Unchanged | 94.31 | 96.58 | 95.93 | Negligible |  |

**Figures**

Figure S1. HPLC-UV chromatogram of blank mobile phase

Figure S2. HPLC-UV chromatogram of the placebo sample

Figure S3. HPLC-UV chromatogram of the standard of tramadol HCl

Figure S4. HPLC-UV chromatogram of the formulation sample of tramadol HCl


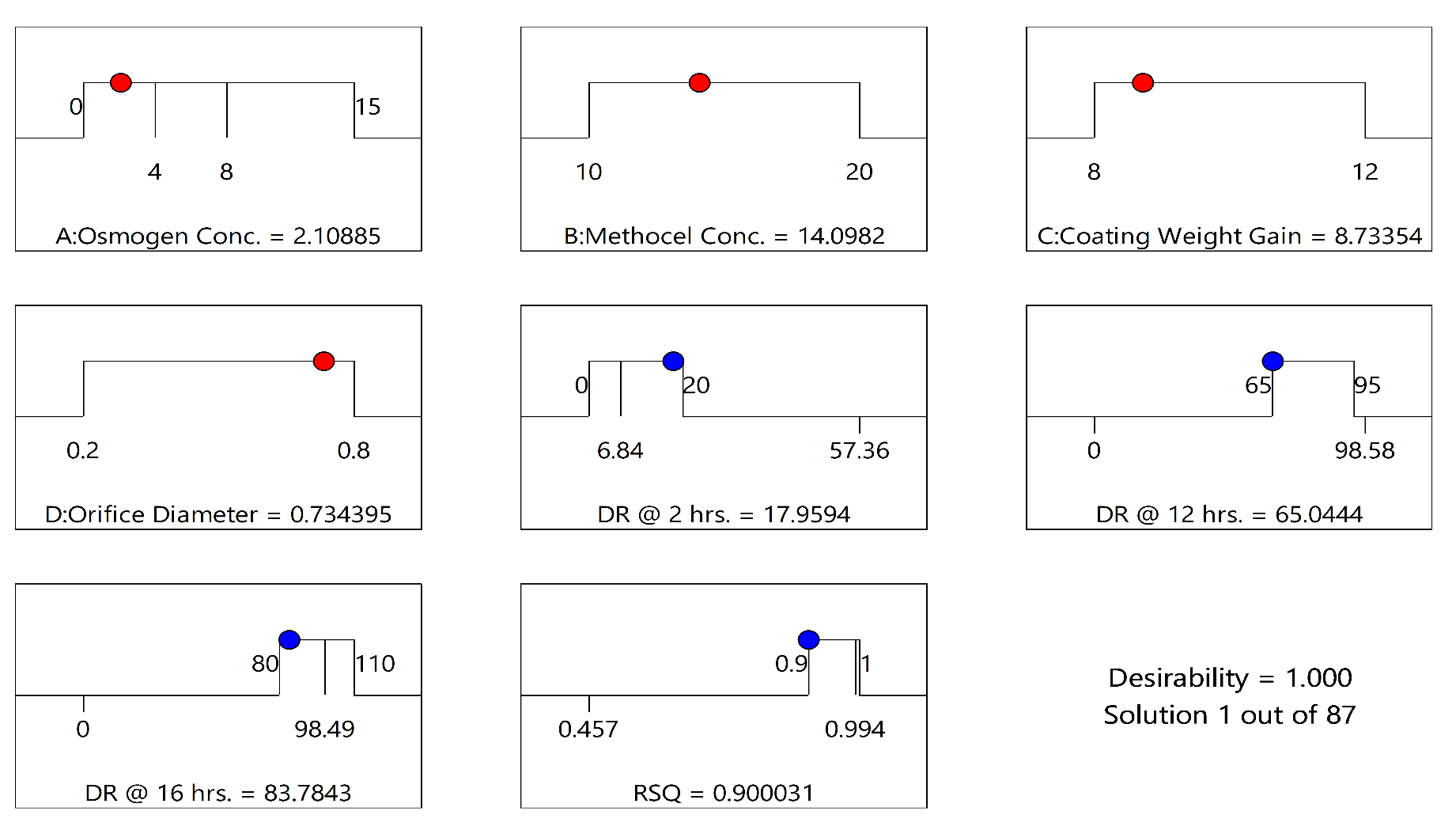


Figure S5. RAMP plots for first optimized formulation (I)


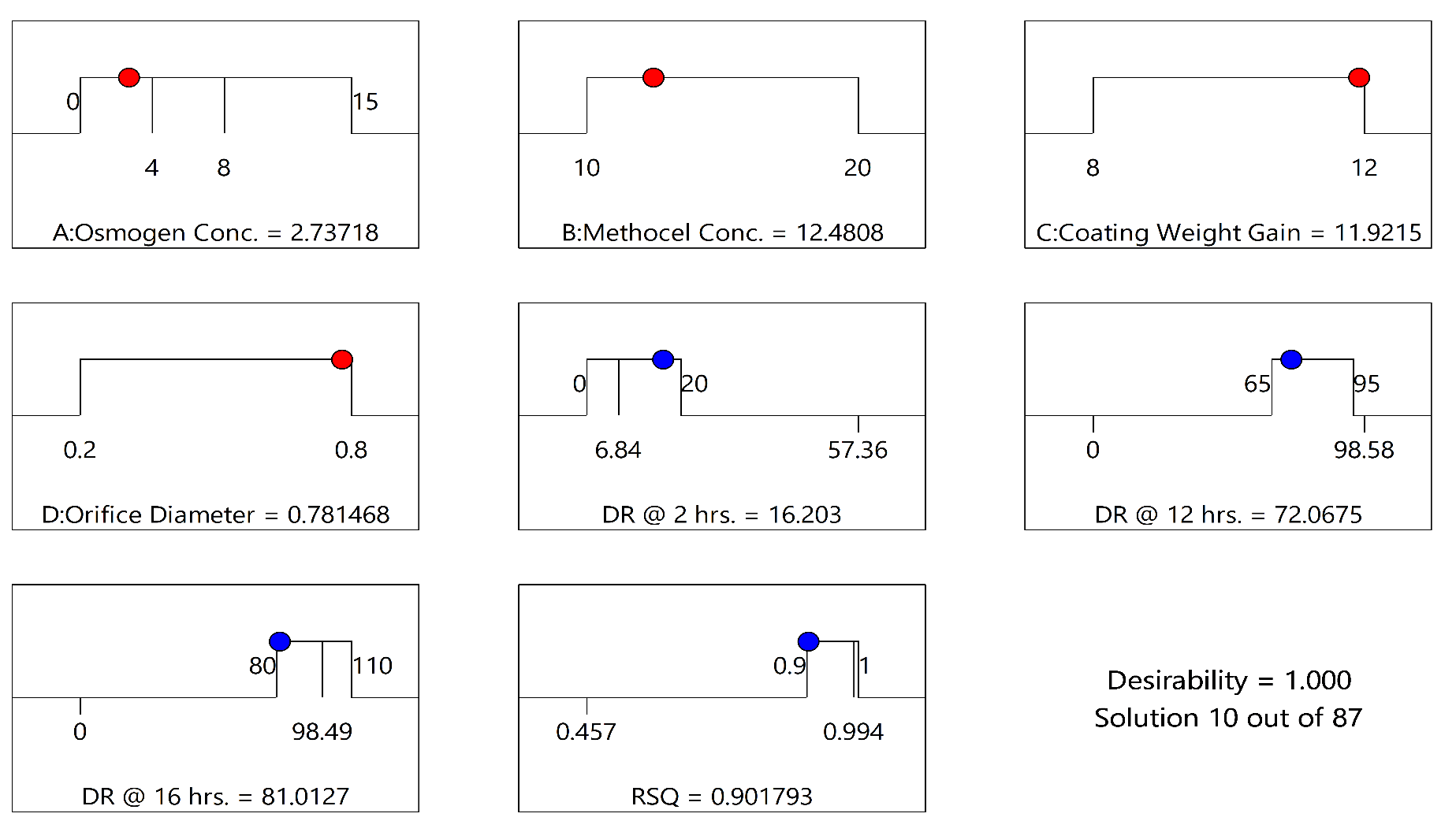


Figure S6. RAMP plots for second optimized formulation (II)


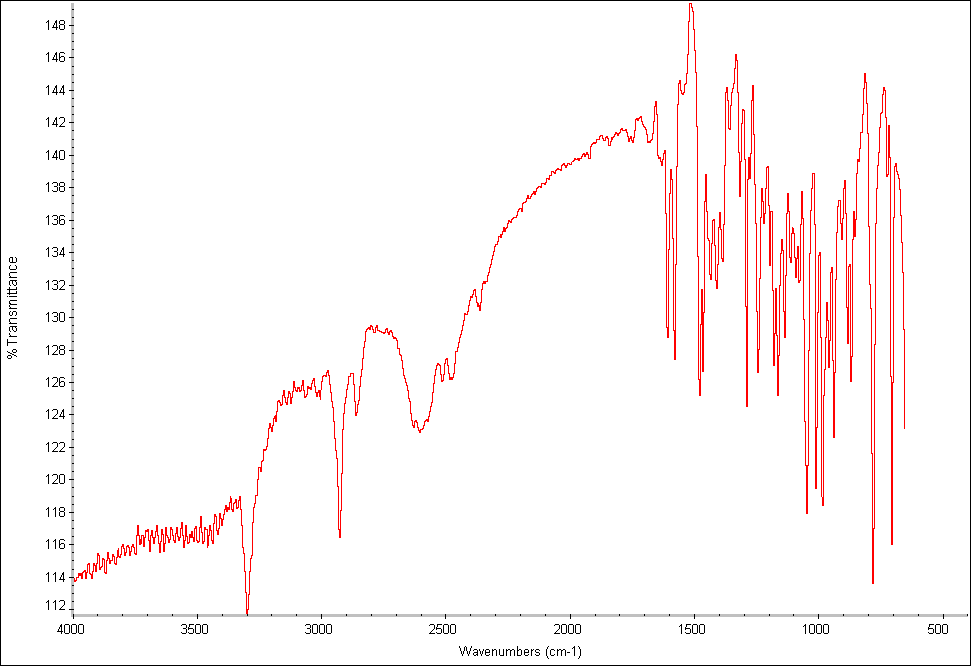


Figure S7. FTIR spectra of tramadol HCl


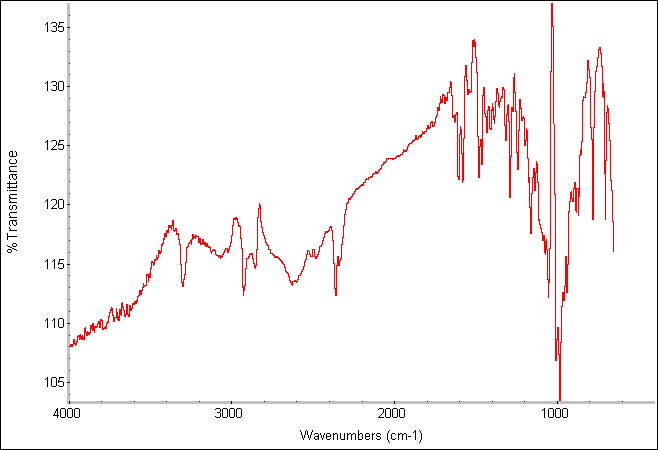


Figure S8. FTIR spectra of formulation F3


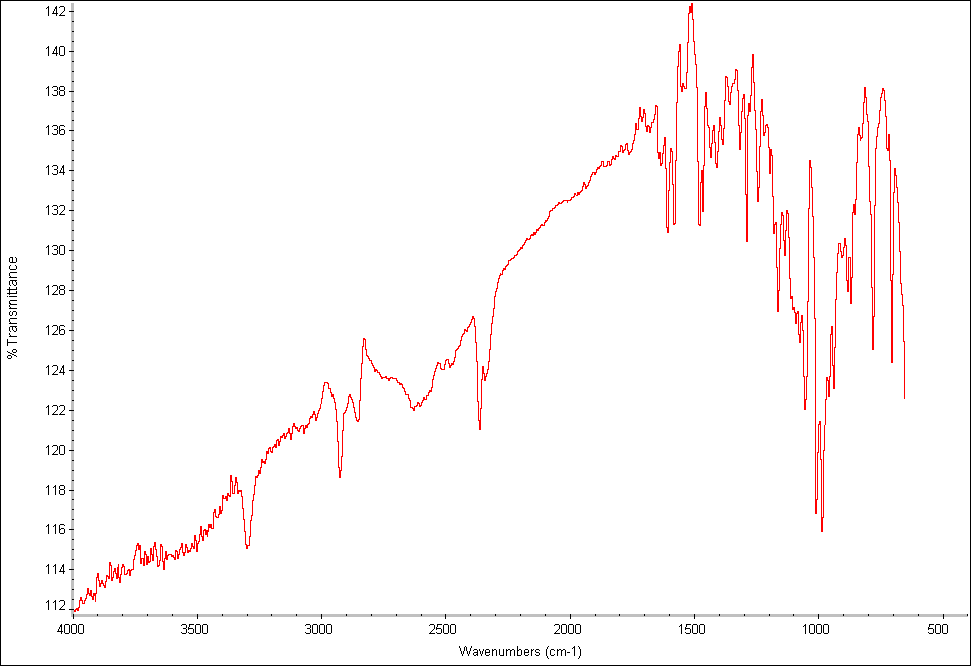


Figure S9. FTIR spectra of formulation F4
